# Tropical gradient descent

**DOI:** 10.1007/s10898-025-01533-1

**Published:** 2025-09-22

**Authors:** Roan Talbut, Anthea Monod

**Affiliations:** Department of Mathematics, Imperial College London, London, UK

**Keywords:** Tropical quasi-convexity, Tropical projective torus, Gradient descent, Location problems, Statistical optimization, Phylogenetics, 49J53, 49K99, 14T90, 62R01, 62R30

## Abstract

We propose a gradient descent method for solving optimization problems arising in settings of tropical geometry—a variant of algebraic geometry that has attracted growing interest in applications such as computational biology, economics, and computer science. Our approach takes advantage of the polyhedral and combinatorial structures arising in tropical geometry to propose a versatile method for approximating local minima in tropical statistical optimization problems—a rapidly growing body of work in recent years. Theoretical results establish global solvability for 1-sample problems and a convergence rate matching classical gradient descent. Numerical experiments demonstrate the method’s superior performance compared to classical gradient descent for tropical optimization problems which exhibit tropical convexity but not classical convexity. We also demonstrate the seamless integration of tropical descent into advanced optimization methods, such as Adam, offering improved overall accuracy.

## Introduction

Where algebraic geometry studies geometric properties of solution sets of systems of multivariate polynomials, *tropical geometry* restricts to the case of polynomials defined by linearizing operations, where the “sum” of two elements is their maximum and the “product” of two elements is their sum. Evaluating polynomials with these operations results in piecewise linear functions. Tropical geometry is a relatively young field of pure mathematics established in the 1990s (with roots dating back further) and has recently become an area of active interest in computational and applied mathematics for its relevance to the statistical analyses of phylogenetic trees [1], the problem of dynamic programming in computer science [2], and in mechanism design for two-player games in game theory [3]. Following the identification of the tropical Grassmannian and the space of phylogenetic trees [4] in particular, there has been a surge of research [1, 5–7] which necessitates the development of new computational techniques for optimization in tropical geometric settings.

Training machine learning models and many computational tasks in statistics entail solving optimization problems; this paper is motivated by statistical optimization problems in tropical geometric settings. Early research in tropical statistics focused on optimization problems such as the identification of Fermat–Weber points which, due to the piecewise linear nature of tropical geometry, can be re-framed as a linear program [6, 7]. Unfortunately, the complexity of these linear programs often scales poorly with the size of our dataset, so gradient methods are more commonly implemented in practice [5, 8]. More recently—following the identification of ReLU neural networks and tropical rational functions [9]—more sophisticated neural network optimization problems are being studied in the tropical setting [10, 11], which necessitates the use of gradient methods for optimization in the tropical setting. In this paper, we address the natural question—given the need for gradient methods in tropical data science, how can we tailor classical gradient methods to the tropical setting? We provide a foundational tropical gradient descent framework to solve the optimization problems which arise in the computation of a wide range of tropical statistics, an area of active interest in applied tropical geometry.

The remainder of this paper is organized as follows. The following section contains the necessary foundations of tropical geometry, an introduction to tropical location problems [12], and an overview of the statistical optimization tasks studied in this paper. Section 3 contains the theoretical contributions of this paper; we formulate steepest descent with respect to the tropical norm and present the theoretical guarantees for tropical descent, most notably its convergence for a wide class of tropically quasi-convex functions and a convergence rate of $O(1/\sqrt{m})$. In Section 4, we perform a comprehensive numerical study of our proposed tropical descent method and its performance in the statistical optimization problems of interest. We conclude with a discussion of possible future work on tropical gradient methods in Section 5.

## Background and preliminaries

In this section, we present a summary of tropical geometry and the statistical optimization problems which we study in the tropical setting.

### Tropical geometry

We begin by reviewing the algebraic and geometric structure of the tropical projective torus, the state space for phylogenetic data and the domain of our tropical statistical optimization problems.

#### Definition 1

(Tropical Algebra) The *tropical algebra* is the semiring ${\overline{\mathbb {R}}} = \mathbb {R}\cup \{- \infty \}$ with the addition and multiplication operators—tropical addition and tropical multiplication, respectively—given by

$$\begin{aligned} a \boxplus b = \max \{a,b\}, \quad a \odot b = a+b. \end{aligned}$$

The additive identity is $-\infty$ and the multiplicative identity is 0. Tropical subtraction is not defined; tropical division is given by classical subtraction.

The tropical algebra defined above is sometimes referred to as the *max-plus algebra*; we can define the min-plus algebra analogously, taking $\min \{ a,b \} =: a \oplus b$ as the additive operation on $\mathbb {R}\cup \{ \infty \}$. These are algebraically equivalent under negation. Unless specified, we use the max-plus convention as this is better suited for phylogenetic statistics on the tropical projective torus [4].

#### Definition 2

(Tropical Projective Torus) The $N-1$-dimensional tropical projective torus is a quotient space constructed by endowing $\mathbb {R}^{N}$ with the equivalence relation

1 $$\begin{aligned} \textbf{x} \sim \textbf{y} \Leftrightarrow \exists \, a { \, \in \mathbb {R}} : \, \textbf{x} = a \odot \textbf{y}; \end{aligned}$$

it is denoted by $\mathbb {R}^{ N}/\mathbb {R}\textbf{1}$. The generalized Hilbert projective metric, also referred to as the *tropical metric*, is given by

$$\begin{aligned} d_{\textrm{tr}}(\textbf{x},\textbf{y}) = \max _i {(}x_i-y_i{)} - \min _i {(}x_i - y_i{)} = \max _{i,j} {(}x_i - y_i - x_j + y_j{)}. \end{aligned}$$

This metric is induced by the tropical norm, which is given by

$$\begin{aligned} \Vert \textbf{x}\Vert _{\textrm{tr}} = \max _i x_i - \min _i x_i. \end{aligned}$$

#### Remark 1

**Fig. 1:**
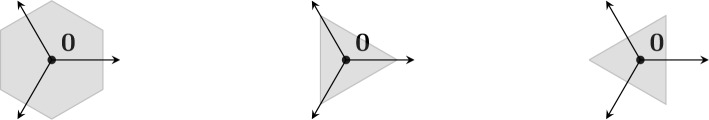
The $d_{\textrm{tr}}$, $d_{\triangle _{\min }}$ and $d_{\triangle _{\max }}$ tropical unit balls in $\mathbb {R}^{3}/\mathbb {R}\textbf{1} \cong \mathcal {H} = \{ \sum x_i = 0 \}$. The solid lines show coordinate directions

**Fig. 2:**
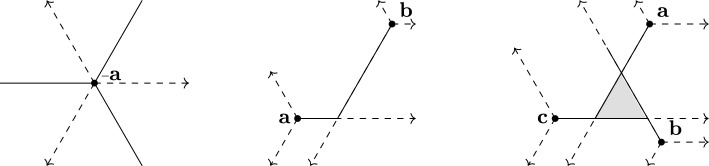
(A tropical hyperplane, tropical line segment and tropical convex hull in $\mathbb {R}^{3}/\mathbb {R}\textbf{1} \cong \mathcal {H} = \{\textbf{x}: \sum x_i = 0 \}$. The dashed lines show coordinate directions

While a single point in the tropical projective torus is given by some ray $\textbf{x} + \mathbb {R}\textbf{1}$, we note that there is a unique representative of this equivalence class whose coordinates sum to zero. In taking such representatives, we can identify the tropical projective torus $\mathbb {R}^{ N}/\mathbb {R}\textbf{1}$ and the hyperplane $\mathcal {H} = \{\textbf{x} \in \mathbb {R}^{ N} : \sum x_i = 0 \}$. Throughout this paper, we use this homeomorphism to visualize the tropical projective torus (e.g., Figures 1 and 2).

The tropical projective torus is the ambient space containing the tropical Grassmannian, which is equivalent to the space of phylogenetic trees [4]. This has sparked the study of tropical data science, formalizing statistical techniques which respect the tropical geometry of the state space [1, 13].

The generalized Hilbert projective metric defined above is widely accepted and preferred for the development of geometric statistical tools on the tropical projective torus [13]. However, recent work has shown theoretical advantages to the use of an asymmetric metric [7].

#### Definition 3

(Tropical Asymmetric Distance) The *tropical asymmetric distances* on $\mathbb {R}^{ N}/\mathbb {R}\textbf{1}$ are given by

$$\begin{aligned}&d_{\triangle _{\min }}(\textbf{a}, \textbf{b}) :=\sum _i (b_i-a_i) - {N}\min _j(b_j-a_j),\\&d_{\triangle _{\max }}(\textbf{a}, \textbf{b}) :={N}\max _j(b_j-a_j) - \sum _i (b_i-a_i). \end{aligned}$$

#### Remark 2

The asymmetric distances act as an $\ell _1$ norm on the tropical projective torus, while the symmetric metric $d_{\textrm{tr}}$ acts as an $\ell _{\infty }$ norm. We note that ${N} d_{\textrm{tr}} = d_{\triangle _{\min }}+d_{\triangle _{\max }}$. Figure 1 shows the balls for each tropical metric of interest.

We now define the primary geometric objects of interest in the tropical projective torus; hyperplanes, lines, and convex hulls. We use the $\max$-convention for these definitions, though we note each has a $\min$-convention equivalent.

#### Definition 4

(Tropical Hyperplane) The *tropical hyperplane* $\mathcal {H}_{\textbf{a}}$ defined by the linear form $a_1 \odot x_1 \boxplus \cdots \boxplus {a_N \odot x_N}$ is given by

$$\begin{aligned} \mathcal {H}_{\textbf{a}} = \{ \textbf{x} { \,\in \mathbb {R}^{N}/\mathbb {R}\textbf{1}} : \exists \, i \ne j\text { s.t. }a_i+x_i=a_j+x_j = \max _k {(}a_k + x_k{)} \}. \end{aligned}$$

#### Definition 5

(Tropical Line Segment) For any two points $\textbf{a}, \textbf{b} \in \mathbb {R}^{ N}/\mathbb {R}\textbf{1}$, the *tropical line segment* between $\textbf{a}$ and $\textbf{b}$ is the set

$$\begin{aligned} \gamma _{\textbf{a} \textbf{b}} = \{ \alpha \odot \textbf{a} \boxplus \beta \odot \textbf{b} \mid \alpha , \beta \in \mathbb {R}\} \end{aligned}$$

with tropical addition taken coordinate-wise.

Tropical line segments define a unique geodesic path between any two points, but general geodesics are not uniquely defined on the tropical projective torus.

#### Definition 6

(Tropical Convex Hull [14]) A set $S \subset \mathbb {R}^{N}/\mathbb {R}\textbf{1}$ is *max-tropically convex* if it contains $\alpha \odot \textbf{x} \boxplus \beta \odot \textbf{y}$ for all $\textbf{x}, \textbf{y} \in S$ and $\alpha , \beta \in \mathbb {R}$. For a finite subset $X = \{\textbf{x}_1,\ldots , \textbf{x}_{{K}}\} \subset \mathbb {R}^{{N}}/\mathbb {R}\textbf{1}$, the *max-plus tropical convex hull* of *X* is the set of all max-tropical linear combinations of points in *X*,

$$\begin{aligned} \text {tconv}_{\max }(X) :=\{ \alpha _1 \odot \textbf{x}_1 \boxplus \cdots \boxplus \alpha _{ K} \odot \textbf{x}_{ K} \mid \alpha _1, \ldots , \alpha _{ K} \in \mathbb {R}\}. \end{aligned}$$

Similarly, the *min-tropical convex hull* of *X*, $\text {tconv}_{\min }(X)$, is the set of all min-tropical linear combinations of points in *X*.

A study of tropically convex functions requires an understanding of both max-tropical and min-tropical convexity, as we will see in our following consideration of tropical location problems.

### Tropical location problems

Tropical location problems are a new avenue of research, motivated by the optimization of statistical loss functions; they involve the minimization over some dataset of some loss function which heuristically increases with distance from some kernel. Here we outline the relevant definitions of tropical location problems and their theoretical behavior as presented by [12]. We introduce the problem of tropical linear regression [15] as motivation.

#### Motivating example

The problem of tropical linear regression looks to find the best-fit tropical hyperplane which minimizes the maximal distance to a set of data points. While this is an inherently statistical optimization problem, it has recently been proven to be polynomial-time equivalent to solving mean payoff games [15].

A tropical hyperplane is uniquely defined by a cone point $\textbf{t} \in \mathbb {R}^{N}/\mathbb {R}\textbf{1}$, allowing us to formulate the best-fit tropical hyperplane as an optimization problem over ${\textbf{t} \in \, } \mathbb {R}^{N}/\mathbb {R}\textbf{1}$.

##### Definition 7

(Tropical Linear Regression [15]) Let $X = \{\textbf{x}_1, \dots , \textbf{x}_K \} \subset \mathbb {R}^{N}/\mathbb {R}\textbf{1}$ be our dataset. The *tropical linear regression problem* finds the vertex $\textbf{t} \in \mathbb {R}^{N}/\mathbb {R}\textbf{1}$ of the best-fit hyperplane:

$$\begin{aligned} \min _{\textbf{t} \in \mathbb {R}^{N}/\mathbb {R}\textbf{1}} f(\textbf{t}) = \max _{k \le K} d_{\textrm{tr}}(\textbf{x}_k, \mathcal {H}_{\textbf{t}}) = \max _{k \le K} { \left( \textstyle \max _i (\textbf{x}_k-\textbf{t})_i - \text {2ndmax}_i (\textbf{x}_k - \textbf{t})_i \right) } \end{aligned}$$

where $\text {2ndmax}_i y_i$ denotes some $y_i$ such that $\forall k \ne i,j: y_j \ge y_i \ge y_k$ such that $\forall \,k$: $y_j \ge y_i \ge y_k$.

This expression for the tropical distance between $\textbf{x}$ and ${\mathcal {H}}_{\textbf{t}}$ was first stated by [16], and has been frequently used in the ongoing study of tropical support vector machines [17, 18].

**Fig. 3:**
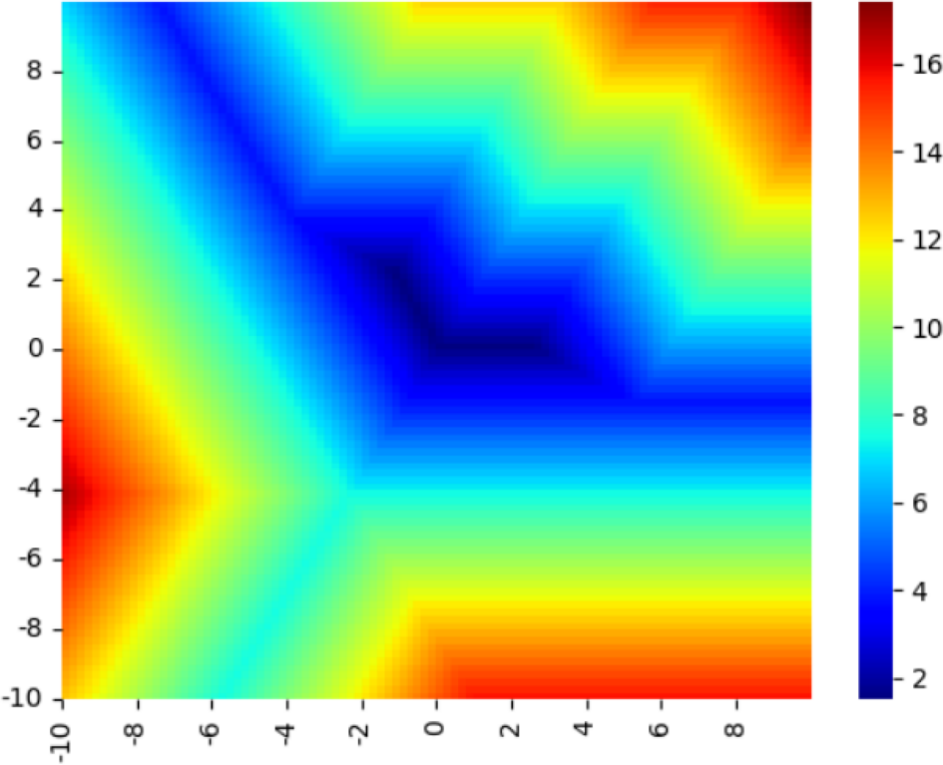
A heat map of a 3-sample linear regression objective on $\mathbb {R}^{3}/\mathbb {R}\textbf{1} \cong \mathcal {H} = \{ \textbf{x}: \sum x_i = 0 \}$

In Figure 3, we see a heatmap of this objective function over the tropical projective plane for a sample of size 3. We see immediately that the objective is not convex. The linear regression objective has sparse gradient almost everywhere, which produces valleys. These properties are considered unfavorable extreme cases for gradient methods, but are particularly common in tropical optimization problems. While the tropical linear regression problem is not convex, it is quasi-convex along tropical line segments; that is, its sub-level sets are tropically convex.

#### Quasi-convexity of tropical functions

In formalizing tropical location problems, the work of [12] first considers functions which increase along tropical geodesics from a kernel $\textbf{v}$; that is, for any point $\textbf{t}$ on a geodesic from $\textbf{v}$ to $\textbf{w}$, we have that $f(\textbf{t}) \le f(\textbf{w})$. This property is equivalent to star-convexity of sub-level sets. To formally state this, we define (oriented) geodesic segments and $\triangle _{\min }$-star convexity of sets.

##### Definition 8

(Oriented Geodesic Segment) The *(oriented) geodesic segment* between $\textbf{a},\textbf{b} \in \mathbb {R}^{ N}/\mathbb {R}\textbf{1}$, is given by $[\textbf{a},\textbf{b}]_{\triangle _{\min }} :=\{ \textbf{x} \in \mathbb {R}^{ N}/\mathbb {R}\textbf{1}: d_{\triangle _{\min }}(\textbf{a},\textbf{x})+ d_{\triangle _{\min }}(\textbf{x},\textbf{b}) = d_{\triangle _{\min }}(\textbf{a},\textbf{b})\}$.

The oriented geodesic segment is the union of all geodesics from $\textbf{a}$ to $\textbf{b}$ with respect to $d_{\triangle _{\min }}$.

##### Definition 9

($\triangle _{\min }$-Star Convex Set) A set $S \subseteq \mathbb {R}^{ N}/\mathbb {R}\textbf{1}$ is a $\triangle _{\min }$-*star-convex* set with kernel $\textbf{v}$ if, for every $\textbf{w} \in S$, we have $[\textbf{v},\textbf{w}]_{\triangle _{\min }} \subseteq S$.

Using the above definitions, we can characterize a loss function which is increasing along tropical geodesics as a function with $\triangle _{\min }$-star-convex sub-level sets. The following definition gives a closed form expression for such functions, while Theorem 1 proves their equivalence.

##### Definition 10

($\triangle _{\min }$-Star-Quasi-Convex Functions [12]) A function $f: \mathbb {R}^{ N}/\mathbb {R}\textbf{1} \rightarrow \mathbb {R}$ is $\triangle _{\min }$-*star-quasi-convex* with kernel $\textbf{v}$ if $f(\textbf{x}) = \hat{\gamma }(\textbf{x}-\textbf{v})$ for some $\gamma : \mathbb {R}^{ N}_{\ge 0} \rightarrow \mathbb {R}$ which is increasing in every coordinate, where we define $\hat{\gamma }(\textbf{x}) :=\gamma (\textbf{x} - (\min _i x_i)\textbf{1})$.

##### Theorem 1

(Theorem 17 of [12]) A continuous function $f: \mathbb {R}^{ N}/\mathbb {R}\textbf{1} \rightarrow \mathbb {R}$ is $\triangle _{\min }$-star-quasi-convex with kernel $\textbf{v}$ if and only if all of its non-empty sub-level sets are $\triangle _{\min }$-star convex with kernel $\textbf{v}$.

##### Remark 3

We can define $\triangle _{\max }$-star-quasi-convex functions similarly, but we also note that a function $f(\textbf{t})$ is a $\triangle _{\max }$-star-quasi-convex function if and only if $f(-\textbf{t})$ is a $\triangle _{\min }$-star-quasi-convex function.

These $\triangle _{\min }$-star-convex functions serve as a loss function with respect to a single data point. For example, the tropical metric $d_{\textrm{tr}}$ is both $\triangle _{\min }$ and $\triangle _{\max }$-star-quasi-convex. The distance to a hyperplane $d_{\textrm{tr}}(\textbf{x}, \mathcal {H}_{\textbf{t}})$ is $\triangle _{\max }$-star-quasi-convex in $\textbf{x}$, but $\triangle _{\min }$-star-quasi-convex in $\textbf{t}$.

As a final remark, we note that $\triangle _{\min }$-star-quasi-convex functions are a special case of tropically quasi-convex functions by the following lemma. We limit our theoretical considerations to $\triangle _{\min }$-star-quasi-convex functions to utilize their closed form expression given in Definition 10, however numerical experiments demonstrate strong performance of our methodology for more general tropical quasi-convex functions.

##### Proposition 1

(Proposition 8 of [12]) Any $\triangle _{\min }$-star-convex set is $\min$-tropically convex. Hence, any $\triangle _{\min }$-star-quasi-convex function is *tropically quasi-convex* in that it has tropically convex sub-level sets.

#### Tropical location problems

The $\triangle _{\min }$-star-convex functions defined above are generally a measure of closeness to the kernel $\textbf{v}$, but in minimizing a statistical loss function we consider proximity to a sample of points. A *location problem* [19] measures the closeness to a sample rather than a single kernel.

##### Definition 11

(Min(/max)-Tropical Location Problem [12]) Consider a dataset $X = \{ \textbf{x}_1, \dots , \textbf{x}_K\} \subset \mathbb {R}^{ N}/\mathbb {R}\textbf{1}$. For $k \le K$, let $h_k$ be a $\triangle _{\min }$-star-quasi-convex function with kernel $\textbf{x}_k$, and let $g: \mathbb {R}^K \rightarrow \mathbb {R}$ be increasing in every coordinate. A *min-tropical location problem* is the minimization of an objective function $f: \mathbb {R}^{ N}/\mathbb {R}\textbf{1} \rightarrow \mathbb {R}$ given by $f = g(h_1, \dots , h_K)$. A *max-tropical location problem* is defined similarly, where the $h_k$ are $\triangle _{\max }$-star-quasi-convex.

The main result of [12] is the following: tropical location problems contain a minimum in the tropical convex hull of its dataset *X*.

##### Theorem 2

(Theorem 20 of [12]) Let *f* be the objective function of a min-tropical location problem. Then there is a minimum of *f* belonging to $\text {tconv}_{\max }(X)$. Conversely, max-tropical location problems have minima belonging to $\text {tconv}_{\min }(X)$.

We can now revisit our motivating example (Definition 7), showing that tropical linear regression is not just a min-tropical location problem, but also demonstrates tropical quasi-convexity in that it has tropically convex sub-level sets.

##### Proposition 2

The tropical linear regression problem is a $\min$-tropical location problem with tropically convex sub-level sets.

##### Proof

$$\begin{aligned} f(\textbf{t})&= {\max _{k \le K} \left( \textstyle \max _i (\textbf{x}_k-\textbf{t})_i - \text {2ndmax}_i (\textbf{x}_k - \textbf{t})_i \right) } \\&\max _{k \le K} \text {2ndmin}_j (\max _i (x_{ki} - t_i) - x_{kj} + t_j) \\&\max _{k \le K} \text {2ndmin}_j (t_j - x_{kj} - \min _i (t_i - x_{ki})) \end{aligned}$$

This is a min-tropical location problem with:

$$\begin{aligned} g(\textbf{h})&= \max _{k \le K} h_k, \\ h_k(\textbf{t})&= \hat{\gamma }(\textbf{t} - \textbf{x}_k), \\ \gamma (\textbf{t})&= \text {2ndmin}_i t_i \end{aligned}$$

This *g* and $\gamma$ are increasing in each coordinate and each $h_k$ has kernel $\textbf{x}_k$, satisfying the conditions for a min-tropical location problem.

As the $h_k$ are $\triangle _{\min }$-star-convex, their sub-level sets are min-tropically convex by Proposition 1. This is preserved when taking a maximum over samples, so *f* also has $\min$-tropically convex sub-level sets. $\square$

As mentioned in Proposition 1, $\triangle _{\min }$-star-quasi-convexity implies tropical quasi-convexity. However, the tropical linear regression problem is a case in which the reverse implication does not hold; in general, the linear regression loss function is not $\triangle _{\min }$-star-quasi-convex.

### Tropical data science

While young, the field of tropical data science is motivated by the crucial question of statistical learning from the increasing volume of phylogenetic tree data [20]. In contrast to the BHV [21] or Robinson–Foulds metrics [22], the tropical interpretation of tree space provides both interpretable geometry and computational efficiency [1, 23]. Many of the statistical methods defined for the tropical setting are framed as optimization problems [5], and in this subsection we review some such tropical statistical optimization problems which we use to test our tropical descent methodology. We note the varying degrees of convexity demonstrated by each of them. While we restrict our considerations through the rest of this paper to statistics which arise from tropical location problems, in Appendix A we discuss other tropical statistical optimization problems.

#### Centrality statistics

Our first problems of interest, Fermat–Weber points and Fréchet means, are centrality statistics for data on a general metric space; Fermat–Weber points [24] act as a generalized median, while Fréchet means [25] are a generalization of the mean.

##### Definition 12

(Tropical Fermat–Weber Points [6]) Let $X = \{\textbf{x}_1, \dots , \textbf{x}_K \} \subset \mathbb {R}^{N}/\mathbb {R}\textbf{1}$ be our dataset. A solution to the following optimization problem is a *tropical Fermat–Weber point*:

$$\begin{aligned} \min _{\textbf{t} \in \mathbb {R}^{N}/\mathbb {R}\textbf{1}} f(\textbf{t}) = \frac{1}{K}\sum _{k \le K} d_{\textrm{tr}}(\textbf{x}_k, \textbf{t}). \end{aligned}$$

##### Definition 13

(Tropical Fréchet means [1]) Let $X = \{\textbf{x}_1, \dots , \textbf{x}_K \} \subset \mathbb {R}^{N}/\mathbb {R}\textbf{1}$ be our dataset. A solution to the following optimization problem is a *tropical Fréchet mean*:

$$\begin{aligned} \min _{\textbf{t} \in \mathbb {R}^{N}/\mathbb {R}\textbf{1}} f(\textbf{t}) = \left[ \frac{1}{K}\sum _{k \le K} d_{\textrm{tr}}(\textbf{x}_k, \textbf{t})^2 \right] ^{1/2}. \end{aligned}$$

The Fermat–Weber and Fréchet mean problems are both max-tropical and min-tropical location problems, as the tropical metric $d_{\textrm{tr}}$ is both $\triangle _{\min }$ and $\triangle _{\max }$-star-quasi-convex. In fact, they are also both classically convex and hence act as a benchmark against which we can test our gradient methods.

#### Wasserstein projections

The next statistical optimization problem was motivated by [26], and looks to identify a tropical projection which minimizes the Wasserstein distance between samples in different tropical projective tori.

##### Definition 14

(Tropical Wasserstein Projections [27]) Let $X = \{ \textbf{x}_1, \dots , \textbf{x}_K \} \subset \mathbb {R}^{N}/\mathbb {R}\textbf{1}$ be a dataset in one space, and $Y = \{ \textbf{y}_1, \dots , \textbf{y}_K \} \subset \mathbb {R}^{M}/\mathbb {R}\textbf{1}$ be a dataset in a different space. Let $J_1, \dots , J_M$ be a non-empty partition of [*N*]. Then the *tropical p-Wasserstein projection problem* is the minimization of the following objective function:

$$\begin{aligned} \min _{\textbf{t} \in \mathbb {R}^{N}/\mathbb {R}\textbf{1}} f_p(\textbf{t}) = \left( \frac{1}{K}\sum _{k \le K} \Vert (\max _{i \in J_j} (\textbf{x}_k - \textbf{t})_i )_{ {j \le M}} - \textbf{y}_k\Vert _{\textrm{tr}}^p\right) ^{1/p} \end{aligned}$$

The *tropical *$\infty$*-Wasserstein projection problem* is the minimization of:

$$\begin{aligned} \min _{\textbf{t} \in \mathbb {R}^{N}/\mathbb {R}\textbf{1}} f_{\infty }(\textbf{t}) = \max _{k \le K} \Vert ( \max _{i \in J_j} (\textbf{x}_k - \textbf{t})_i )_{ {j \le M}} - \textbf{y}_k\Vert _{\textrm{tr}} \end{aligned}$$

In contrast to the work by [28] which looks to solve the optimal transport problem for intrinsic measures on $\mathbb {R}^{N}/\mathbb {R}\textbf{1}$, this *p*-Wasserstein projection problem is computing an optimal projection for empirical samples.

##### Proposition 3

The tropical $p,\infty$-Wasserstein projections are min-tropical location problems, and the $\infty$-Wasserstein projection has $\min$-tropically convex sub-level sets.

##### Proof

Let $\textbf{z}_k = (x_{ki} - y_{kj})_{i \le N}$ where *j* is such that $J_j$ is the unique partition set containing *i*. We then have that

$$\begin{aligned} \Vert (\max _{i \in J_j} (\textbf{x}_k - \textbf{t})_i )_{ {j \le M}} - \textbf{y}_k\Vert _{\textrm{tr}}&=\max _{b} \max _{a \in J_b} ( z_{ka} - t_a ) - \min _{j} \max _{i \in J_j} (z_{ki} - t_i ) \\&= \max _{a} (z_{ka} - t_a ) + \max _j\min _{i \in J_j} ( t_i - z_{ki} ) \\&= \max _j (\min _{i \in J_j} (t_i - z_{ki} - \min _{a} (t_a - z_{ka} {)))} \end{aligned}$$

The $p, \infty$-Wasserstein projections can then be written as

$$\begin{aligned} f_{p} (\textbf{t})&=\left( \frac{1}{K}\sum _{k \le K} \left( \max _j (\min _{i \in J_j} (t_i - z_{ki} - \min _{a} (t_a - z_{ka} {)))} \right) ^p\right) ^{1/p} \\ f_{\infty } (\textbf{t})&=\max _{k \le K} \max _j (\min _{i \in J_j} (t_i - z_{ki} - \min _{a} (t_a - z_{ka}{)))} \end{aligned}$$

These are max-tropical location problems with:

$$\begin{aligned} g_{p}(\textbf{h})&= \left( \frac{1}{K}\sum _{k \le K} h_k^p\right) ^{1/p}, \\ g_{\infty }(\textbf{h})&= \max _{k \le K} h_k, \\ h_k(\textbf{t})&= \hat{\gamma }(\textbf{t} - \textbf{z}_k), \\ \gamma (\textbf{t})&= \max _j\min _{i \in J_j}t_i. \end{aligned}$$

The $g_p, g_{\infty }$ and $\gamma$ are increasing in each coordinate and each $h_k$ has kernel $\textbf{z}_k$, satisfying the conditions for a min-tropical location problem.

As the $h_k$ are $\triangle _{\min }$-star-convex, their sub-level sets are min-tropically convex by Proposition 1. This is preserved when taking a maximum over samples, so $f_{\infty }$ also has $\min$-tropically convex sub-level sets. $\square$

**Fig. 4:**
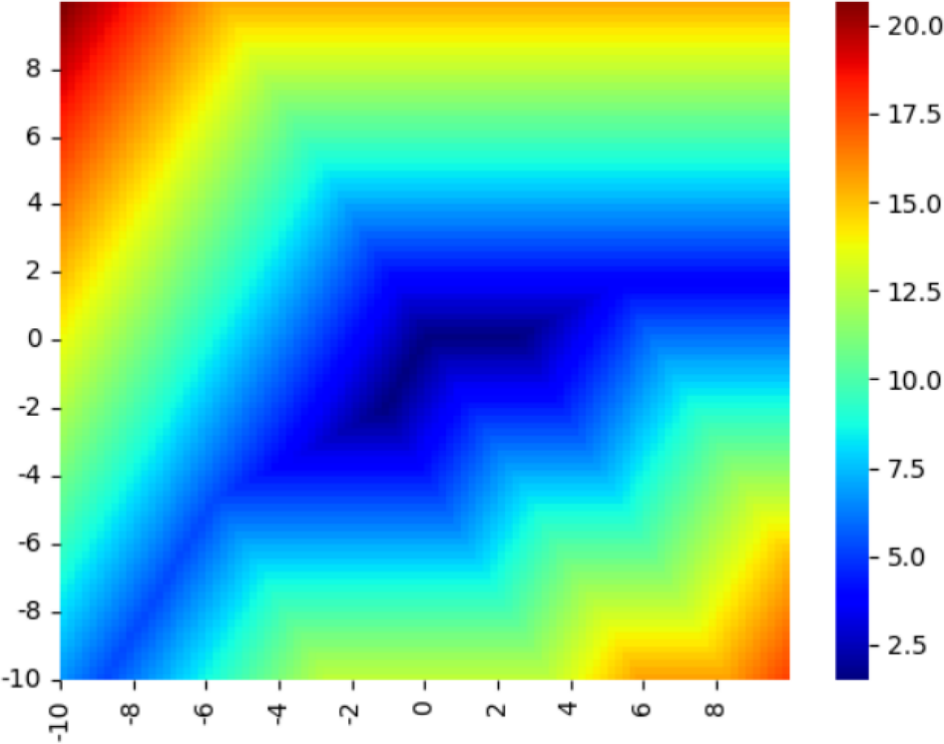
A heat map of a 3-sample $\infty$-Wasserstein projection objective on $\mathbb {R}^{3}/\mathbb {R}\textbf{1} \cong \mathcal {H} = \{ \textbf{x}: \sum x_i = 0 \}$

Figure 4 shows a heatmap for the $\infty$-Wasserstein projection objectives. As in the tropical linear regression problem, it is piecewise linear, non-convex and has sparse gradient.

## Steepest descent with the tropical norm

This section comprises the theoretical contributions of this paper; we outline our proposed method of tropical descent, and the theoretical convergence guarantees for tropical descent when applied to tropical location problems.

We treat optimization over $\mathbb {R}^{N}/\mathbb {R}\textbf{1}$ as optimization over $\mathbb {R}^{N}$ with the knowledge that *f* is constant along $\textbf{1}$-rays. We assume we can compute some gradient-like function $\nabla f: \mathbb {R}^N \rightarrow \mathbb {R}^N$ which satisfies Assumption 1.

### Assumption 1

Let $f = g(h_1, \dots , h_K)$ be a min-tropical location problem. Then we have that

$$\begin{aligned} \nabla f(\textbf{t})_j < 0 \Rightarrow \exists \, k \text { s.t. } j \in \text {argmin}_i(t_i - x_{ki}). \end{aligned}$$

This is a very general assumption, which holds for some of the most natural gradient-like functions. For example, if $\nabla f(\textbf{t})$ is given by some Clarke gradient, that is, some element of the *generalized Clarke derivative* of *f*, then Lemma 1 shows that Assumption 1 holds.

### Definition 15

(Generalized Clarke Derivative (2.13, I.I of [29])) We define the set $M_f(\textbf{t})$ by

$$\begin{aligned} M_f(\textbf{t}) = \{ \textbf{z} \in \mathbb {R}^N : \nabla f(\textbf{y}_l) \rightarrow \textbf{z}, \text { where }\textbf{y}_l \rightarrow \textbf{t}, f\text { differentiable at each }\textbf{y}_l \}. \end{aligned}$$

Then the *generalized Clarke derivative* is given by

$$\begin{aligned} \partial f(\textbf{t}) = \text {conv}M_f(\textbf{t}). \end{aligned}$$

### Lemma 1

Suppose $f = g(h_1, \dots , h_K)$ is min-tropical location problem, and for all $\textbf{t}$, $\nabla f(\textbf{t})$ is some element of $\partial f(\textbf{t})$. Then Assumption 1 holds.

### Proof

Suppose that $\nabla f(\textbf{t})_j < 0$. Then there is a sequence of differentiable $\textbf{y}_\ell$ satisfying $\nabla f(\textbf{y}_\ell )_j < 0$, $\textbf{y}_\ell \rightarrow \textbf{t}$. Hence by the chain rule, as *g* is increasing in every $h_k$, there is some $h_k$ which is strictly decreasing in the $j^{th}$ coordinate at $\textbf{y}_\ell$. WLOG, the same $h_k$ is strictly decreasing in the $j^{th}$ coordinate for all $\textbf{y}_\ell$. As $\gamma$ is increasing in every coordinate, $h_k = \hat{\gamma }(\textbf{y}_\ell - \textbf{x}_k)$ is strictly decreasing in the $j^{th}$ coordinate if and only if $j \in \text {argmin}_i (y_{\ell i} - x_{ki})$. This is preserved through the limit as $\ell \rightarrow \infty$, so we conclude that $j \in \text {argmin}_i (t_i - x_i )$. $\square$

In practice, rather than computing elements of the generalized Clarke derivative, we use PyTorch automatic differentiation [30]. The derivatives computed by PyTorch are dependent on the computational representation of a function, but are designed to respect the chain rule. We must therefore ensure that our computational representation of *g* has non-negative derivatives in each $h_k$, while each $h_k$ is computed such that its $t_j$ derivative is negative only if $j \in \text {argmin}_i (t_i - x_{ki})$. For such a computational representation of *f*, Assumption 1 will hold by the chain rule. For all the tropical location problems we consider in this paper, we find their most natural computational representation gives rise to derivatives $\nabla f$ satisfying Assumption 1.

### Proposed method

In defining tropical descent, we refer back to the motivation behind classical gradient descent—steepest descent directions.

#### Definition 16

(Steepest Descent Direction [31]) A normalized *steepest descent direction* at $\textbf{t}$ with respect to the norm $\Vert \cdot \Vert$ is:

$$\begin{aligned} \textbf{d} = \text {argmin}\{ \nabla f(\textbf{t})^{\top } \textbf{v} : \Vert \textbf{v}\Vert = 1 \}. \end{aligned}$$

With respect to the Euclidean norm, the normalized derivative is a steepest descent direction. The steepest descent directions under the tropical norm are characterized by the following lemma.

#### Lemma 2

Suppose $\nabla f \ne \textbf{0}$. Then the direction $\textbf{v}$ is a normalized tropical steepest descent direction (up to $\sim$ equivalence) iff

$$\begin{aligned} \nabla f_i < 0&\Rightarrow v_i = 1, \\ \nabla f_i > 0&\Rightarrow v_i = 0 \end{aligned}$$

#### Proof

WLOG, we assume $0 \le v_i \le 1$. Then

$$\begin{aligned} \nabla f^{\top } \textbf{v} \ge \sum _{i:\,\nabla f_i < 0} \nabla f_i \end{aligned}$$

This is achieved if and only if $v_i = 1$ for all *i* such that $\nabla f_i <0$, and $v_i = 0$ for all *i* such that $\nabla f_i >0$. We note that any such $v_i$ satisfies $\Vert \textbf{v}\Vert _{\textrm{tr}} = 1$ as $\textstyle \sum _i \nabla f_i = 0$ by the equivalence relation of the tropical projective torus, and $\nabla f_i \ne \textbf{0}$ so $\nabla f$ has positive and negative coordinates. $\square$

Lemma 2 uniquely defines a descent direction $\textbf{d}$ if and only if, for every coordinate *i*, $\nabla f_i \ne 0$. To uniquely define a descent direction for any $\textbf{t}$, we take the direction $\textbf{d}$ given by

$$\begin{aligned} d_i = {\left\{ \begin{array}{ll} 1 &  \text { if }\nabla f_i < 0, \\ 0 &  \text { otherwise.} \end{array}\right. } \end{aligned}$$

We refer to this as a *min-tropical descent* direction. In contrast,

$$\begin{aligned} d_i = {\left\{ \begin{array}{ll} -1 &  \text { if }\nabla f_i > 0, \\ 0 &  \text { otherwise.} \end{array}\right. } \end{aligned}$$

defines a *max-tropical descent* direction.

Throughout the rest of this paper, we use the min-tropical descent direction as convention, and will specify when the max-tropical descent direction is used instead. Tropical descent therefore refers to the method outlined by Algorithm 1, for some specified step size sequence $(a_{ m})_{ m\ge 1}$, while we refer to classical gradient descent as classical descent.

**Algorithm 1:**
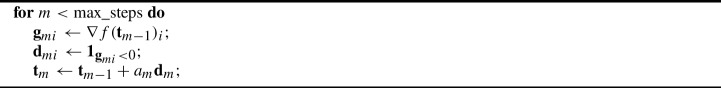
Tropical Descent

We note that over a large number of steps, Algorithm 1 can produce large $t_{ mi}$ values as each coordinate is non-decreasing in *m*. If this results in overflow or precision errors, a normalization operation can be included in each step, such as subtracting $\frac{1}{N} \sum _{{i \le N} } t_{ mi}$ from each $t_{ mi}$. The function *f* is invariant under such a translation due to the equivalence relation on the tropical projective torus.

In [5, 8], classical descent is implemented for the computation of Fermat–Weber points; this is the most natural choice as the Fermat–Weber problem is classically convex. However, the linear regression and Wasserstein projection problems lack classical convexity, and hence do not consistently produce accurate solutions via classical descent. It is these tropically convex problems for which tropical descent is necessary.

### Convergence guarantees

In this section we prove the theoretical guarantees of tropical descent for min-tropical location problems. We show that tropical descent must converge to the tropical convex hull of the data, and hence will necessarily find a minimum of a $\triangle _{\min }$-star-convex problem. Throughout this section, we assume the step sizes $a_{ m}$ satisfy $a_{ m} \rightarrow 0, \sum a_{ m} = \infty$.

We first highlight the key difference in stability between classical descent and tropical descent; if some component of the derivative is locally zero, tropical descent cannot converge in that neighborhood.

#### Proposition 4

Let $\textbf{t}_{ m}$ be some sequence defined by tropical descent. Assume that for all *m*, $\nabla f(\textbf{t}_{ m}) \ne \textbf{0}$. Suppose in some bounded open *U*, $\nabla f_i$ is identically 0. Then no point in *U* is a convergence point for $\textbf{t}_{ m}$; in particular, if $\textbf{t}_m \in U$ there is some $n > m$ such that $\textbf{t}_n \notin U$.

#### Proof

Assume otherwise. Then for all $n \ge m$, we have $\nabla f(\textbf{t}_n)_i = 0$, so $t_{ni}$ is constant. At each step, we increase at least one coordinate by $a_n$, so as $n \rightarrow \infty$:

$$\begin{aligned} \sum _j t_{nj} \ge \sum _{j} t_{mj} + \sum _{n \ge m' \ge m} a_{m^{'}} \rightarrow \infty \end{aligned}$$

Therefore $\Vert \textbf{t}_n\Vert _{\textrm{tr}}$ will become arbitrarily large, as $\max _j t_{nj} \rightarrow \infty$ while $\min _j t_{nj} \le t_{mi}$. Then we must have left *U*; contradiction. $\square$

As a result of the proposition above, local minima such as the valleys in Figures 3 and 4 can be stable with respect to classical descent but unstable with respect to tropical descent.

We next prove the main convergence result of this work; tropical descent must converge to the tropical convex hull of the data points.

#### Theorem 3

Suppose *f* is a min-tropical location problem with respect to the dataset $X = \{\textbf{x}_1, \dots , \textbf{x}_K \}$. Let $(\textbf{t}_{ m})_{ m \ge 1}$ be a sequence of points defined by tropical descent, such that for all *m*: $\nabla f(\textbf{t}_{ m}) \ne 0$. Let $V = \text {tconv}_{\max }(\textbf{x}_1, \dots , \textbf{x}_K)$, and define

$$\begin{aligned} \Delta _j(\textbf{t}) = \sum _{ {k \le K}} \sum _{{i \le N}} [t_j - x_{kj} - t_i + x_{ki}]^+. \end{aligned}$$

For any $\epsilon > 0$, let $M_1$ be such that for all $m \ge M_1$, $a_m < \epsilon /N$. Let $M_2$ be such that for all $m \ge M_2$ $s_{m} \ge \max _j \Delta _j(\textbf{t}_{M_1}) + s_{M_1} - K\epsilon$, where $s_m$ is the partial sum of the step size sequence $a_m$. Then for all $m \ge M_2$ we have:

$$\begin{aligned} d_{\textrm{tr}}(\textbf{t}_{ m}, V) \le \epsilon . \end{aligned}$$

#### Proof

For each $i \le N$, we define the functions

$$\begin{aligned} \delta _j(\textbf{t}) = \min _{ {k \le K}} \left[ \sum _{ {i \le N}} [t_j - x_{kj} - t_i + x_{ki}]^+ \right] . \end{aligned}$$

$$\square $$

#### Claim 1

For all $\textbf{t} \in \mathbb {R}^{N}/\mathbb {R}\textbf{1}$, we have $d_{\textrm{tr}}(\textbf{t},V) \le \max _j \delta _j(\textbf{t})$.

#### Proof

As discussed in [2], there is a well-defined metric projection map $\pi _V$ onto *V* given by:

$$\begin{aligned}  &   \pi _V(\textbf{t}) = \lambda _1 \odot \textbf{x}_1 \boxplus \dots \boxplus \lambda _K \odot \textbf{x}_K, \\  &   \quad \text { where }\lambda _k = \max \{ \lambda \in \mathbb {R}: \, \lambda \odot \textbf{x}_{k} \boxplus \textbf{t} = \textbf{t} \} = \min _i(t_i - x_{ki}). \end{aligned}$$

The distance from $\textbf{t}$ to *V* is then given by $d(\textbf{t}, \pi _V(\textbf{t}))$, and we have:

$$\begin{aligned} d(\textbf{t}, V)&= d(\textbf{t}, \pi _V(t)) \\&= \Vert (\max _k (\min _i (t_i - x_{ki}) - t_j + x_{kj})_{j \le N} \Vert _{ \textrm{tr}}. \end{aligned}$$

We note that for all *j*, *k*, the term $\min _i (t_i - x_{ki}) - t_j + x_{kj}$ is negative. Hence:

$$\begin{aligned} d(\textbf{t}, V)&\le 0 - \min _j (\max _k (\min _i (t_i - x_{ki}) - t_j + x_{kj})) \\&= \max _j (\min _k (t_j - x_{kj} - \min _i (t_i - x_{ki}))) \\&\le \max _j \delta _j(\textbf{t}). \end{aligned}$$

The claim is proved. $\square$

We will now prove that for all $m \ge M_2$, we have that $\delta _j(\textbf{t}_m) \le \epsilon$.

#### Claim 2

If $\delta _i(\textbf{t}_m) = 0$ and $m \ge M_1$, then $\delta _i(\textbf{t}_{m+1}) < \epsilon$.

#### Proof

For all ${k \le K, j \le N}$, we have that $t_{mj} - x_{kj} - t_{mi} + x_{ki}$ is 1-Lipschitz in $\textbf{t}$ (with respect to $d_{\textrm{tr}}$). Therefore $\delta _j$ is *N*-Lipschitz in $\textbf{t}$. By the tropical descent rule:

$$\begin{aligned} \delta _j(\textbf{t}_{m+1})&\le \delta _j(\textbf{t}_m) + Nd_{\textrm{tr}}(\textbf{t}_{m}, \textbf{t}_{m+1})\\&= 0 + Na_{m+1} \\&< \epsilon . \end{aligned}$$

The claim is proved. $\square$

#### Claim 3

If $$\delta _j(\textbf{t}_m) > 0$$ then $$\delta _j(\textbf{t}_{m+1}) \le \delta _j(\textbf{t}_{m})$$.

#### Proof

Suppose $\delta _j(\textbf{t}_m) > 0$. Then $\forall k$, $t_{mj} - x_{kj} > \min _i (t_{mi} - x_{ki})$, so by Lemma 1 we have $\nabla f_j \ge 0$. The tropical steepest descent direction therefore has $d_{mj} = 0$. We conclude that

$$\begin{aligned} \forall \, k,i : \quad&t_{(m+1)j} - x_{kj} - t_{(m+1)i} + x_{ki} \le t_{mj} - x_{kj} - t_{mi} + x_{ki}, \\&\Rightarrow \quad \delta _j(\textbf{t}_{m+1}) \le \delta _j(\textbf{t}_m). \end{aligned}$$

The claim is proved. $\square$

#### Claim 4

For some $$M_1 \le m \le M_2$$, $$\delta _j(\textbf{t}_m) \le \epsilon $$.

#### Proof

We note that $K\delta _j(\textbf{t}) \le \Delta _j(\textbf{t})$; hence if $\Delta _j(\textbf{t}_{M_1}) < K\epsilon$ then we are done.

We assume $\delta _j(\textbf{t}_m) > \epsilon$ for all $M_1 \le m \le M_2$, and prove the result by contradiction. Then as in the proof of the previous claim, at each step we have $\nabla f_j \ge 0$, $d_{mj} = 0$ and $t_{mj}$ is fixed for $M_1 \le m \le M_2$.

We have assumed that for each *m*, $\nabla f \ne \textbf{0}$ and so there is some *i* such that $\nabla f_i < 0$, and by Lemma 1, there is some *k* such that $i \in \text {argmin}_{\ell } ( t_{m\ell } - x_{k\ell })$. Hence:

$$\begin{aligned} \forall \, \ell : \quad t_{mj} - x_{kj} - t_{mi} + x_{ki}&\ge t_{mj} - x_{kj} - t_{m\ell } + x_{k\ell }, \\ \Rightarrow \quad t_{mj} - x_{kj} - t_{mi} + x_{ki}&\ge \delta _j(\textbf{t}_m)/ N, \\&> \epsilon /N, \\&\ge a_m. \end{aligned}$$

Hence this term in the summation of $\Delta _j(\textbf{t}_m)$ decreases by $a_m$, while other terms are non-increasing as $t_{mj}$ is fixed.

Noting that $K\delta _j(\textbf{t}) \le \Delta _j(\textbf{t})$, we conclude

$$\begin{aligned} K\delta _j(\textbf{t}_{M_2})&\le \Delta _j (\textbf{t}_{M_2}), \\&\le \Delta _j (\textbf{t}_{M_1}) - s_{M_2} + s_{M_1}, \\&\le K\epsilon . \end{aligned}$$

Contradiction. So the claim is proved. $\square$

Finally, from the claims above, we conclude that $\forall m \ge M_2, {i \le N}$, we have $\delta _i(\textbf{t}_m) \le \epsilon$, and so by our first claim, we have $d_{\textrm{tr}}(\textbf{t}_m, V) \le \epsilon$. $\square$

As proven in [12], tropical location problems have minima in the tropical convex hull of the data. The theorem above says that according to tropical descent, only such minima can be stable. We cannot do much better in terms of convergence results for general location problems; they can have disconnected sub-level sets so we would not expect gradient methods to find a global minimum. We would require some degree of convexity to guarantee global solvability, which is exactly what the following corollary gives us; tropical descent will necessarily converge to a minimum of any $\triangle _{\min }$-star-quasi-convex function.

#### Corollary 1

Let $f(\textbf{t})$ be a $\triangle _{\min }$-star-quasi-convex function, and let $(\textbf{t}_{ m})_{ m \ge 1}$ be a sequence of points defined by tropical descent such that for all *m*: $\nabla f(\textbf{t}_{ m}) \ne 0$. Then there is some global minimum $\textbf{t}^*$ such that for all $m \ge M_2$, $d_{\textrm{tr}}(\textbf{t}_m, \textbf{t}^*) \le \epsilon$ where $M_2$ is as in Theorem 3.

This corollary follows from considering the 1-sample case of tropical location problems; corollary 1 establishes convergence to the kernel of a $\triangle _{\min }$-star-quasi-convex function, which must necessarily be a global minimum. This convergence result holds even if *f* does not have a unique minimum — it may be that *f* has multiple global minima but a unique kernel, or *f* may have a full dimensional set of global minima upon which $\nabla f(\textbf{t}) = \textbf{0}$ and tropical descent terminates.

As a further corollary, we note that we can achieve the same result for max-tropical location problems by using max-tropical descent directions.

#### Corollary 2

Suppose *f* is a max-tropical location problem with respect to the dataset $X = \{\textbf{x}_1, \dots , \textbf{x}_K \}$. Let $(\textbf{t}_{ m})_{ m \ge 1}$ be a sequence of points defined via max-tropical descent directions, such that for all *m*: $\nabla f(\textbf{t}_{ m}) \ne 0$. Let $V = \text {tconv}_{\min }(\textbf{x}_1, \dots , \textbf{x}_K)$, and define

$$\begin{aligned} \Delta '_i(\textbf{t}) = \sum _{{k \le K}} \sum _{{j \le N}} [t_j - x_{kj} - t_i + x_{ki}]^+. \end{aligned}$$

For any initial $\textbf{t}_0$ and $\epsilon > 0$, let $M_1$ be such that for all $m \ge M_1$, $a_m \le \epsilon /N$. Let $M_2$ be such that for all $m \ge M_2$ $s_{m} \ge \max _i \Delta '_j(\textbf{t}_{M_1}) + s_{M_1} - K\epsilon$. Then for all $m \ge M_2$ we have:

$$\begin{aligned} d_{\textrm{tr}}(\textbf{t}_{ m}, V) \le \epsilon . \end{aligned}$$

#### Remark 4

The results above indicate convergence to *V* or $\textbf{t}^*$ in the tropical topology of $\mathbb {R}^{N}/\mathbb {R}\textbf{1}$, but $\textbf{t}_m$ cannot not converge in the Euclidean topology of $\mathbb {R}^N$ due to the divergent partial sums of $a_n$.

We conclude by establishing a foundational bound for the convergence rate of tropical descent; we take step sizes given by $a_m = \alpha m^{-1/2}\Vert \nabla f(\textbf{t}_m) \Vert _{\textrm{tr}}$, and assume derivatives have bounded tropical norm as is the case for piecewise linear 1-Lipschitz objective functions (with finitely many linear pieces). In this case, we prove convergence at a rate of $O(1/\sqrt{m})$.

#### Proposition 5

Suppose *f* is a min-tropical location problem as in Theorem 3. Assume that there is some $L \in \mathbb {N}$ such that for all $\textbf{t}_m$, $1/L \le \Vert \nabla f(\textbf{t}_m)\Vert _{\textrm{tr}} \le 2$, and assume further there is some *D* such that $\forall m \in \mathbb {N}: \, \sum _{k \le K} d_{\triangle _{\max }}(\textbf{x}_k, \textbf{t}_{m}) \le KD$. We define $a_m = \alpha m^{-1/2}\Vert \nabla f(\textbf{t}_m) \Vert _{\textrm{tr}}$. Then for all *m* satisfying

2 $$\begin{aligned} { m \ge \max \left\{ \left( \sqrt{2} + \frac{KDL}{2\alpha } - \sqrt{LKN-1} \right) ^2-1, \left( \frac{2 \alpha N}{D}\right) ^2 \right\} , } \end{aligned}$$

we have that:

$$\begin{aligned} d_{\textrm{tr}}(\textbf{t}_m, V) \le {\frac{2 \sqrt{2} \alpha N}{\sqrt{m+1} - KDL/2\alpha + \sqrt{LKN-1} - \sqrt{2}} } = \epsilon _m. \end{aligned}$$

In particular, if *f* is a $\triangle _{\min }$-star-quasi-convex function, then there is some global minimum $\textbf{t}^*$ such that

$$\begin{aligned} d_{\textrm{tr}}(\textbf{t}_m, \textbf{t}^*) \le {\frac{2 \sqrt{2} \alpha N}{\sqrt{m+1} - DL/2\alpha + \sqrt{LN-1} - \sqrt{2}} = \epsilon '_m.} \end{aligned}$$

In order to use Theorem 3, we will need to find some $M_1$ such that $a_{M_1} < \epsilon /N$ (our step size is small enough) and $s_{m} \ge \max _j \Delta _j({\textbf{t}}_{M_1}) + s_{M_1} - K\epsilon$ (we have taken enough steps to get close to *V*). Our condition that *m* is large enough such that $\sqrt{m+1} - KDL/2\alpha + \sqrt{LKN-1} \ge \sqrt{2}$ ensures that such an $M_1$ exists, while $\sqrt{m} \ge 2 \alpha N/D$ ensures that $M_1 \le m$. We can then take the largest such $M_1$ to ensure that the corresponding $\epsilon$ is as tight as possible.

#### Proof

We fix *m* satisfying Eq. (2) and define:

$$\begin{aligned} {\xi (m) }&{:=\frac{\sqrt{m+1}}{2} - \frac{KDL}{4\alpha } + \sqrt{\left( \frac{\sqrt{m+1}}{2} - \frac{KDL}{4\alpha }\right) ^2 + LKN-1},} \\ M_1&:=\left\lfloor \xi (m)^2 \right\rfloor . \end{aligned}$$

We first note that by the quadratic mean inequality and as $\sqrt{m+1} - KDL/2\alpha + \sqrt{LKN-1} \ge \sqrt{2}$:

$$\begin{aligned} \xi (m)&= \frac{\sqrt{m+1}}{2} - \frac{KDL}{4\alpha } + \sqrt{\left( \frac{\sqrt{m+1}}{2} - \frac{KDL}{4\alpha }\right) ^2 + LKN-1} \\&\ge \frac{\sqrt{m+1}}{2} - \frac{KDL}{4\alpha } + \frac{1}{\sqrt{2}} \left( \frac{\sqrt{m+1}}{2} - \frac{KDL}{4\alpha } + \sqrt{LKN-1} \right) \\&\ge \frac{1}{\sqrt{2}} \left( \sqrt{m+1} - \frac{KDL}{2\alpha } + \sqrt{LKN-1} \right) \ge 1. \end{aligned}$$

Therefore we have that

$$\begin{aligned} {1} \le \sqrt{M_1} \le \xi (m), \end{aligned}$$

and so $\sqrt{M_1}$ lies between the roots of the polynomial

$$\begin{aligned} { p(t) = L^{-1}t^2 + \left( KD/2\alpha - L^{-1}\sqrt{m+1}\right) t+ L^{-1} - KN}. \end{aligned}$$

Therefore:

$$\begin{aligned} {L^{-1}}(M_1+1) + \left( {KD /2\alpha } - {L^{-1}} \sqrt{m+1}\right) \sqrt{M_1}- KN \le 0, \\ {L^{-1}}\sqrt{M_1}\sqrt{M_1+1} + {KD\sqrt{M_1}/2\alpha } - {L^{-1}}\sqrt{M_1}\sqrt{m+1} \le KN, \\ {KD} + 2\alpha L^{-1}(\sqrt{M_1+1} - \sqrt{m+1}) \le \frac{2\alpha KN}{\sqrt{M_1}}. \end{aligned}$$

We now show that $M_1 \le m$. Suppose otherwise; then by the inequality above:

$$\begin{aligned} KD&\le \frac{2 \alpha K N}{\sqrt{M_1}} + 2\alpha L^{-1}(\sqrt{m+1} - \sqrt{M_1+1}) \\&< \frac{2 \alpha K N}{\sqrt{m}} \end{aligned}$$

This contradicts our assumption that $m \ge (2\alpha N/D)^2$, so we conclude that $M_1 \le m$. $\square$

#### Claim 5

For all $n \ge {m}$ we have $a_n \le \epsilon _m / N$.

#### Proof

We have shown that $\xi (m) \ge 1$, so:

$$\begin{aligned} M_1&\ge \xi (m)^2 - 1, \\&\ge \left( \xi (m) -1\right) ^2, \\ \sqrt{M_1}&\ge \xi (m) - 1, \\&\ge {\frac{1}{\sqrt{2}} \left( \sqrt{m+1} - \frac{KDL}{2\alpha } + \sqrt{LKN-1} \right) - 1}. \end{aligned}$$

Hence, by the step size definition and the fact that $n \ge m \ge M_1$:

$$\begin{aligned} a_n&\le \frac{2 \alpha }{\sqrt{M_1}}, \\&\le {\frac{2 \sqrt{2} \alpha }{\sqrt{m+1} - KDL/2\alpha + \sqrt{LKN-1} - \sqrt{2}}} = \epsilon _m/N. \end{aligned}$$

The claim is proved. $\square$

#### Claim 6

We have $$\max _j \Delta _j(\textbf{t}_{M_1}) + s_{M_1} - s_m \le K\epsilon _m $$.

#### Proof

We note that

$$\begin{aligned} \Delta _j(\textbf{t}_{M_1})&= \sum _{k \le K} \sum _{i \le N} [t_{M_1j} -x_{kj} - t_{M_1i} + x_{ki}]^+,\\&\le \sum _{k \le K} d_{\triangle _{\max }}(\textbf{x}_k, \textbf{t}_{M_1}),\\&\le KD. \end{aligned}$$

And therefore we have $\max _j \Delta _j(\textbf{t}_{M_1}) \le KD$. The partial sums are bounded by:

$$\begin{aligned} s_{M_1} - s_m&\le \sum _{n=M_1+1}^m -a_n \\&\le \sum _{n=M_1+1}^m \frac{-\alpha }{Ln^{1/2}} \\&\le \sum _{n=M_1+1}^m - 2 \alpha L^{-1} (\sqrt{n+1} - \sqrt{n}) \\&\le 2\alpha L^{-1} {(\sqrt{M_1+1} - \sqrt{m+1})}. \end{aligned}$$

Therefore:

$$\begin{aligned} \max _i\Delta _i(\textbf{t}_{M_1}) + s_{M_1} - s_m&\le {KD} +2\alpha L^{-1}(\sqrt{M_1+1} - \sqrt{m+1}), \\&\le \frac{2\alpha KN}{\sqrt{M_1}} \le K\epsilon _m. \end{aligned}$$

$$\square $$

Hence by Theorem 3, we have that

$$\begin{aligned} d_{\textrm{tr}}(\textbf{t}_m, V) \le \epsilon _m. \end{aligned}$$

The bound for the tropically quasi-convex case follows from Corollary 1. $\square$

Our error bound in Proposition 12 is a useful foundational result on the convergence behavior of tropical descent; for sufficiently nice problems, tropical descent will converge at a rate of $O(1/\sqrt{m})$. However, a more detailed study of the numerical analysis of tropical descent would be valuable. We outline possible questions below, as well as presenting a comparison of our error bounds with tropical descent for tropical linear regression.

Firstly, the constant term $LD/2\alpha$ in the denominator of $\epsilon _m$ will typically be larger than necessary, resulting in a weaker error bound. In particular, this constant term is quick to dominate for larger problems. Further work improving these constant factors would produce much tighter and more robust error bounds. Secondly, the error $\epsilon _m$ bounds the distance to a tropical convex hull *V*, which is rarely a useful quantity to bound. We would rather use $\epsilon '_m$, which bounds the distance to a global minimum, but our tropical location problems are not known to be $\triangle _{\min }$-star-quasi-convex. It would be particularly useful to generalize our error bound $\epsilon _m'$ to such problems which exhibit some local (but not global) $\triangle _{\min }$-star-quasi-convexity.

For a qualitative view on Proposition 5, we compare $\epsilon _m'$ and the convergence rate of tropical descent for tropical linear regression. As noted, tropical linear regression is tropically quasi-convex (Proposition 2) but not known to be $\triangle _{\min }$-star-quasi-convex, so does not meet the assumptions of Proposition 5; despite this, we see $\epsilon _m'$ is often an effective upper bound on the error in $\textbf{t}$.

**Fig. 5:**
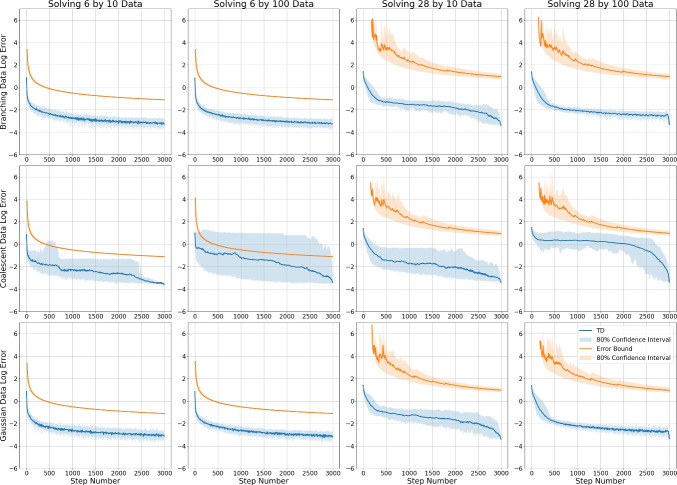
The mean log error bound $\textrm{log}\, \epsilon _m'$ and the mean log tropical distance to the estimated minimum $\textbf{t}^*$ when applying tropical descent (TD) with $\alpha = 1$ to solve the tropical linear regression problem. For each dataset, we take 50 random initialization points and compute $\textrm{log}\, \epsilon _m'$ and $\textrm{log}\, d_{\textrm{tr}}(\textbf{t}_m, \textbf{t}^*)$ independently, then take an average. We also record the 10th and 90th percentile values to estimate an 80% confidence interval

We perform tropical descent for tropical linear regression using 12 different datasets; these are generated via branching processes, coalescent processes, or a Gaussian distribution on $\mathbb {R}^{6}/\mathbb {R}\textbf{1}$ and $\mathbb {R}^{28}/\mathbb {R}\textbf{1}$. We consider sample sizes of 10 or 100. These are the same datasets used in Section 4, and further details on their generation can be found in Appendix B. We run computations using a learning rate of $\alpha = 1$, for steps up to $m \le 3000$. The tropical linear regression problem has $\Vert \nabla f(\textbf{t}_m)\Vert = 2$ almost everywhere, so we take $L=1$ when computing $\epsilon _m'$. We estimate $\textbf{t}^*$ to be the mean of the final 10 steps of tropical descent (TD), and compute a tight estimate for *D* by taking

$$\begin{aligned} D\approx \max _{m \le 3000} d_{\triangle _{\max }} (\textbf{t}^*,\textbf{t}_m). \end{aligned}$$

Fig. 5 shows the mean log error bound $\epsilon _m'$ with the mean log tropical distance to $\textbf{t}^*$.

Examining Fig. 5, we first note that the error $\epsilon _m'$ is orders of magnitude smaller for the lower dimensional problems, which is primarily a result of the constant terms in the denominator of $\epsilon _m'$. As we have noted, it would be of significant value to reduce these constant terms in future numerical analytical studies of tropical descent. These constant terms are also the cause of noise in the mean error bound for high dimensional problems, as we require more steps for the error to be well-defined. We also note that although $\epsilon _m'$ is a reasonable upper bound for the distance to $\textbf{t}^*$ in many cases, this upper bound does not hold for the low dimensional coalescent datasets. It is unclear whither this is due to poor estimates for *D* and $\textbf{t}^*$, or due to tropical linear regression not being $\triangle _{\min }$-star-quasi-convex. We conclude by noting that we are observing a convergence rate of $O(1/\sqrt{m})$ for most experiments, with this rate appearing particularly distinctly for branching process and Gaussian data.

## Numerical experiments

In this section we compare the practical performance of classical descent, tropical descent, SGD, tropical SGD, Adam, Adamax, and tropical Adamax, with a focus on their stability behavior. The implementation for all these experiments is available in the GitHub repository at https://github.com/Roroast/TropicalGradDescent.

### Methods

We begin by outlining our implementation of steepest descent, stochastic gradient descent (SGD), and Adam as well as their tropical variants. We take a heuristic approach to the tropicalization of SGD and Adam, replacing derivatives with tropical steepest descent directions in each case.

**Steepest Descent.** We will perform classical descent (CD) and tropical descent (TD) using equivalent step size sequences $a_m$ for both methods, which we fix as

$$\begin{aligned} a_{ m} = \frac{\gamma \Vert \nabla f(\textbf{t}_{ m}) \Vert }{\sqrt{ m}}, \end{aligned}$$

where $\Vert \cdot \Vert$ is the Euclidean norm for classical descent and the tropical norm for tropical descent. When $\nabla f(\textbf{t})$ is bounded this is a well-behaved step rule; $a_{ m}$ will converge to 0. Furthermore, for the piecewise linear problems, $\Vert \nabla f(\textbf{t}_{ m})\Vert$ is bounded away from 0 almost everywhere which is not a global minimum, so $\sum a_{m} = \infty$.

**Stochastic Gradient Descent.** Stochastic gradient descent is a variant of gradient descent which looks to improve computational speed by approximating $\nabla f(\textbf{t})$; rather than computing the loss and its derivative at $\textbf{t}$ with respect to the entire dataset $f(\textbf{t}; X)$, we evaluate the loss and its derivative with respect to a random sample $\textbf{x}_{ m} \in X$, $f(\textbf{t}; \textbf{x}_{ m})$. This is simplified with the notation $f_{ m}(\textbf{t})$. As with gradient descent, we use the step size sequence given by

$$\begin{aligned} a_{ m} = \frac{\gamma \Vert \nabla f_{ m}(\textbf{t}_{ m}) \Vert }{\sqrt{ m}}, \end{aligned}$$

where $\Vert \cdot \Vert$ is the Euclidean norm for stochastic gradient descent (SGD) and the tropical norm for tropical stochastic gradient descent (TSGD).

**Adam Variants.** The Adam algorithm [32] uses gradient momentum to compound consistent but small effects. This is done by taking first and second moments of the gradient over all steps using exponentially decaying weights. The Adamax algorithm is a variant of the Adam algorithm motivated by the use of the $\infty$-moments rather than second moments.

To adapt the Adam algorithm for the tropical setting, we take moments of an unnormalized tropical descent direction. When looking to take higher moments of the gradient over *m*, the $L_{\infty }$ is the natural option for the tropical setting. We therefore look to use the Adamax recursion relation on the $\infty$-moment estimate $\textbf{u}_{ m}$:

$$\begin{aligned} \textbf{u}_{ m+1} = \max (\beta _2 \textbf{u}_{ m}, \textbf{d}_{ m}). \end{aligned}$$

The tropicalized Adamax algorithm is then given by Algorithm 2.

**Algorithm 2:**
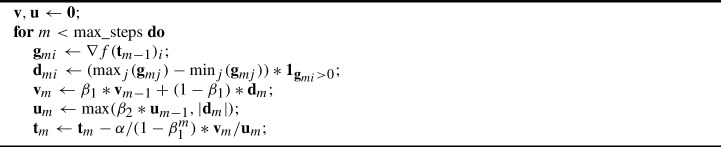
TrAdamax

### Computation

We now look to solve our centrality statistics, Wasserstein projection problems, and tropical linear regression using the 7 gradient methods discussed: classical descent (CD), tropical descent (TD), stochastic gradient descent (SGD), tropical stochastic gradient descent(TSGD), Adam, Adamax, and TrAdamax. We run computations for 12 datasets of different sizes, shapes and dimensions; motivated by phylogenetic data science applications, we consider datasets generated by branching processes, coalescent processes, or Gaussian distributions on $\mathbb {R}^{N}/\mathbb {R}\textbf{1}$, for $N = 6, 28$ and samples of size 10 or 100. We take the same random initializations for each gradient method, taken from a Gaussian distribution on $\mathbb {R}^{N}$. Appendix B provides further details on the simulated datasets and computational methodology used. For each gradient method, we use learning rates which have been optimized for each dataset size and dimension, across different distribution types (Appendix C).

Rather than investigating the relative convergence rates of each method, we are interested in the relative stability of local minima. We therefore plot the CDF of the relative log error after 1000 steps over 50 random initializations, to reveal concentrations of error values where multiple initializations stabilize. When the CDF of one method dominates another, it has a higher probability of achieving the same error or less and is therefore a more suitable method.

As we will observe, tropical gradient methods are stable at fewer points than classical gradient methods; as a result, tropical methods typically have further to travel before reaching a stable point. Over few steps, this would be compensated for by selecting larger learning rates for tropical gradient methods relative to classical gradient methods, leading to less precise results in the case of tropical gradient methods. Our computations are run over 1000 steps, as this gives enough time for tropical gradient methods to stabilize, given our initializations, without sacrificing learning rate; we see in Appendix C that neither classical nor tropical learning rates are consistently greater than the other.

#### Centrality statistics

We first consider the behavior of tropical descent methods for Fermat–Weber points and Fréchet means. These optimization problems are classically convex problems, as well as being both $\max$-tropical and $\min$-tropical location problems, and therefore lend themselves well to gradient methods. The experimental behavior of these problems is similar, so in this section we include the results for the Fermat–Weber problem while the results for Fréchet means can be found in Appendix E.

**Fig. 6:**
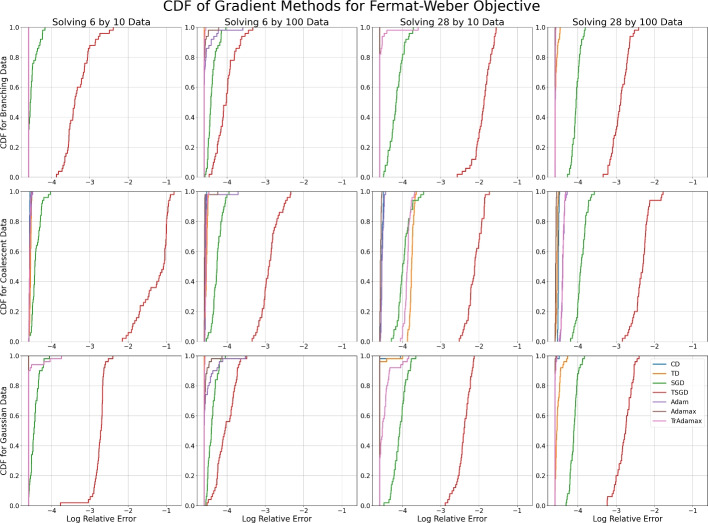
The CDF of the log relative error after 1000 steps for each gradient method with tuned learning rates across 50 random (Gaussian) initializations when minimizing the Fermat–Weber objective. Each subfigure corresponds to a dataset with sample size $K=10,100$ of dimensionality $N=6,28$, sampled from a branching process, coalescent process, or a Gaussian distribution

Figure 6 shows the CDF of the log relative error after 1000 steps of each gradient method for the 12 datasets. We note that the deterministic methods achieve similar results for most datasets, while the stochastic methods achieve the worst error for all 12 datasets and TSGD is performing worse than SGD. The disparity between SGD and TSGD is greatest when the dimensionality of the data is greater than the size of the dataset, and for coalescent data. We see similar behavior in the log error of tropical descent and TrAdamax; they perform on par with their classical counterparts for each dataset other than the 28 by 10 coalescent data, for which the tropical gradient methods are not achieving the same accuracy (Table 1). While it is unclear why this is, we note that coalescent data is supported on the space of ultrametric trees, which is a tropical hyperplane [33, 34] and may influence the stability of minima.

**Table 1:** The mean log relative error of each gradient method after 1000 steps with tuned learning rates for branching process, coalescent process, and Gaussian datasets of size $K=10,100$ in $\mathbb {R}^{6}/\mathbb {R}\textbf{1}$, $\mathbb {R}^{28}/\mathbb {R}\textbf{1}$ when minimizing the Fermat–Weber objective function. The minimal mean errors for each dataset are in bold

Data	CD	TD	SGD	TSGD	Adam	Adamax	TrAdamax
6$\times$10 Branching Data	**−4.60**	**−4.60**	−4.50	−3.32	**−4.60**	**−4.60**	**−4.60**
6$\times$10 Coalescent Data	**−4.58**	−4.55	−4.40	−1.28	−4.57	−4.57	−4.57
6$\times$10 Gaussian Data	**−4.60**	**−4.60**	−4.44	−2.75	**−4.60**	**−4.60**	−4.56
6$\times$100 Branching Data	**−4.60**	**−4.60**	−4.39	−4.02	−4.54	−4.58	−4.59
6$\times$100 Coalescent Data	−4.55	−4.54	−4.25	−2.88	−4.56	**−4.58**	−4.55
6$\times$100 Gaussian Data	**−4.60**	**−4.60**	−4.39	−4.03	−4.52	−4.57	−4.59
28$\times$10 Branching Data	**−4.60**	**−4.60**	−4.15	−1.89	**−4.60**	**−4.60**	−4.56
28$\times$10 Coalescent Data	−4.53	−3.76	−3.96	−2.11	−4.52	**−4.57**	−3.87
28$\times$10 Gaussian Data	−4.58	−4.58	−4.07	−2.40	**−4.60**	**−4.60**	−4.47
28$\times$100 Branching Data	**−4.59**	−4.57	−4.04	−2.89	**−4.59**	**−4.59**	**−4.59**
28$\times$100 Coalescent Data	−4.51	−4.54	−3.91	−2.33	−4.39	**−4.58**	−4.39
28$\times$100 Gaussian Data	**−4.59**	−4.53	−4.10	−2.78	**−4.59**	**−4.59**	**−4.59**

#### Wasserstein projections

**Fig. 7:**
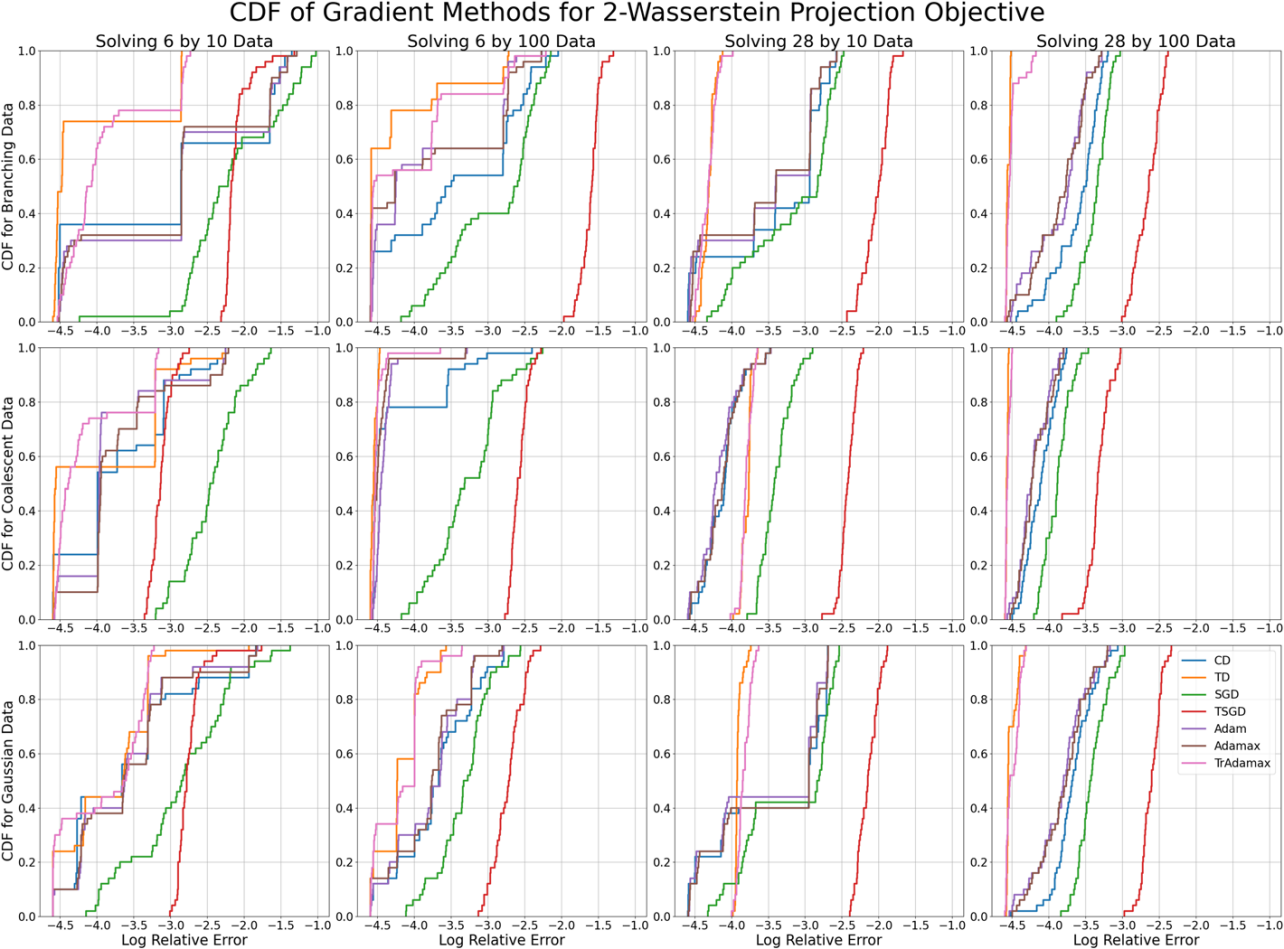
The CDF of the log relative error after 1000 steps for each gradient method with tuned learning rates across 50 random (Gaussian) initializations when minimizing the 2-Wasserstein projection objective. Each subfigure corresponds to a dataset with sample size $K=10,100$ of dimensionality $N=6,28$, sampled from a branching process, coalescent process, or a Gaussian distribution

**Fig. 8:**
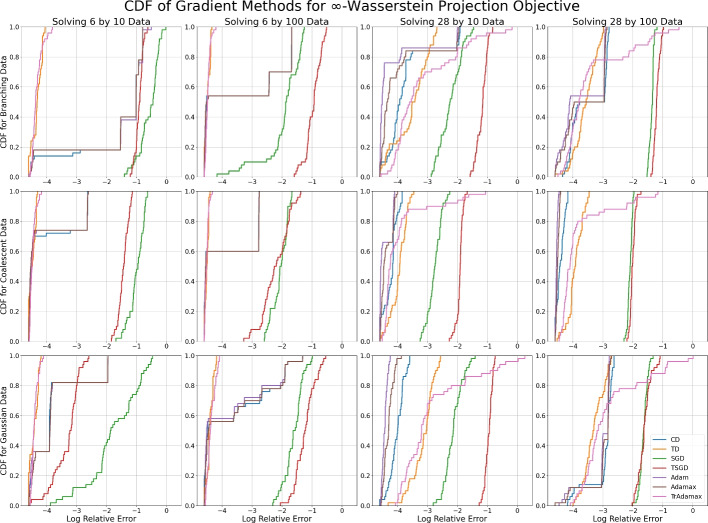
The CDF of the log relative error after 1000 steps for each gradient method with tuned learning rates across 50 random (Gaussian) initializations when minimizing the $\infty$-Wasserstein projection objective. Each subfigure corresponds to a dataset with sample size $K=10,100$ of dimensionality $N=6,28$, sampled from a branching process, coalescent process, or a Gaussian distribution

The Wasserstein projection problems highlight the differences in stability behavior between classical and tropical gradient methods, as they are $\min$-tropical location problems but not classically convex. The CDFs of the final log relative errors can be found in Figures 7 and 8.

In Figures 7 and 8 we see large steps at various intervals in the CDF plots, which is indicative of stable local minima. This is particularly prevalent in low dimensional space. Classical descent, Adam, and Adamax are particularly susceptible to this, while tropical descent and TrAdamax are more likely to pass these local minima. This difference is most pronounced when solving the $\infty$-Wasserstein problem in low dimensions—we are almost certain to reach the minimum with relative error $\le e^{-3}$, while classical methods have a 50-80% chance of achieving the same error.

#### Linear regression

**Fig. 9:**
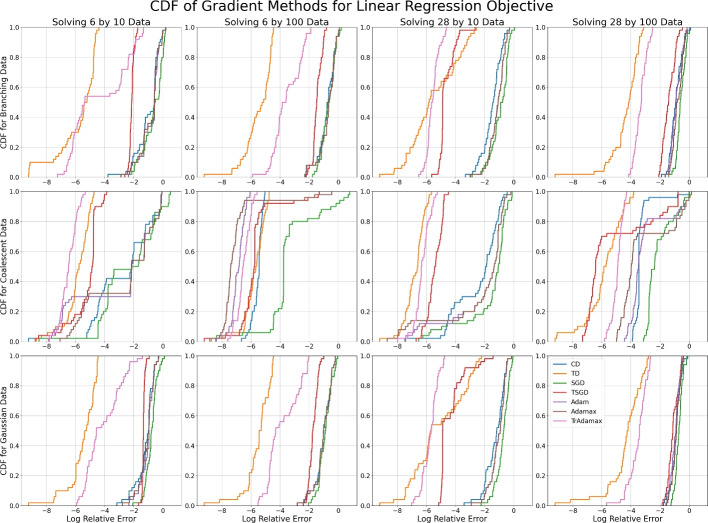
The CDF of the log error after 1000 steps for each gradient method with tuned learning rates across 50 random (Gaussian) initializations when minimizing the linear regression objective. Each subfigure corresponds to a dataset with sample size $K=10,100$ of dimensionality $N=6,28$, sampled from a branching process, coalescent process, or a Gaussian distribution

As the coalescent data lies on a tropical hyperplane [33, 34], the minimum of the linear regression function for such data is exactly 0. Figure 9 therefore shows the CDF of the log error rather than the CDF of the log relative error.

**Table 2:** The mean log relative error of each gradient method after 1000 steps with tuned learning rates for branching process, coalescent process, and Gaussian datasets of size $K=10,100$ in $\mathbb {R}^{6}/\mathbb {R}\textbf{1}$, $\mathbb {R}^{28}/\mathbb {R}\textbf{1}$ when minimizing the linear regression objective function. The minimal mean errors for each dataset are in bold

Data	CD	TD	SGD	TSGD	Adam	Adamax	TrAdamax
6$\times$10 Branching Data	−0.97	**−5.79**	−0.56	−2.16	−0.80	−0.84	−4.39
6$\times$10 Coalescent Data	−2.70	−5.87	−2.11	−5.47	−2.97	−2.66	**−6.58**
6$\times$10 Gaussian Data	−1.19	**−5.48**	−0.80	−1.35	−1.05	−1.10	−4.10
6$\times$100 Branching Data	−0.79	**−5.49**	−0.71	−1.43	−0.72	−0.72	−3.53
6$\times$100 Coalescent Data	−5.54	−5.76	−3.11	−5.57	−6.90	**−7.01**	−6.52
6$\times$100 Gaussian Data	−1.04	**−5.50**	−0.88	−1.70	−1.02	−1.02	−3.81
28$\times$10 Branching Data	−1.49	−5.51	−0.93	−4.49	−1.14	−1.14	**−5.57**
28$\times$10 Coalescent Data	−2.43	**−6.63**	−1.77	−5.55	−2.16	−2.29	−6.18
28$\times$10 Gaussian Data	−1.26	−5.16	−0.79	−4.29	−1.08	−1.08	**−5.72**
28$\times$100 Branching Data	−0.90	**−4.37**	−0.61	−1.42	−0.85	−0.77	−3.32
28$\times$100 Coalescent Data	−3.33	**−5.84**	−2.17	−5.35	−3.02	−3.20	−4.93
28$\times$100 Gaussian Data	−0.91	**−4.35**	−0.62	−1.07	−0.96	−0.90	−3.51

Table 2 shows the mean log errors of each gradient method and dataset, from which we see that the tropical methods outperform their classical counterparts for all but one dataset, often by a factor of two. In Figure 9 we see that the CDF of log errors for tropical descent and TrAdamax consistently dominates those of classical descent, Adam and Adamax, to the extent that the support of their CDFs does not intersect that of their classical counterparts for some datasets. We also note that unlike the previous problems, TSGD is outperforming SGD when solving the linear regression problem, often to the extent that TSGD outperforms all classical gradient methods.

### Tropical statistics for applications

We conclude our computational experiments by using tropical gradient methods to solve tropical statistical problems which appear in phylogenetic data analysis and game theory. In particular we look to use tropical centrality statistics to estimate the species tree of a multi-species coalescent model [35] , and we use tropical linear regression to estimate the hidden preference factors in the Auction model introduced by [15], which is a variant of the first-price sealed-bid auction model [36].

#### Multi-species coalescent model

A *species tree* refers to a rooted metric tree which represents the evolutionary relationships between a set of species. However, for a specific gene, we may not observe the same branching pattern, instead observing a gene-specific evolution which is referred to as a *gene tree*. This raises one of the key questions in phylogenetics; how do we estimate the species tree from a set of gene trees? The multi-species coalescent model is a probabilistic model for gene trees given a certain species tree (see [35] for a detailed introduction), which is particularly valuable for formulating Bayesian estimators for species trees and producing simulated data with which to test species tree estimation methodologies.

**Fig. 10:**
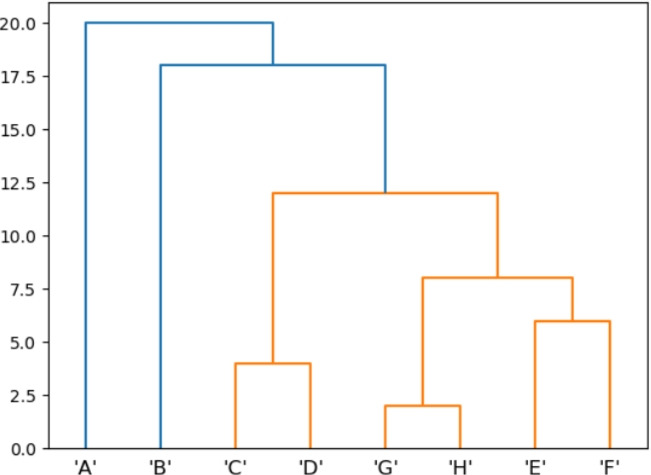
The underlying species tree used for the multi-species coalescent model generating our dataset of gene trees. The topology of this tree can be represented by the Newick string “(((((F,E),(H,G)),(D,C)),B),A)”

In this section, we consider the tropical Fermat-Weber and Fréchet means of gene trees as estimators for the underlying species tree. We fix a species tree with 8 leaves (see Fig. 10), then use Dendropy [37] to sample 100 gene trees from a multi-species coalescent model with this underlying species tree and normalize this dataset to have average tropical norm 1.

We then compute a Fermat-Weber point and Fréchet mean for this dataset using each of our gradient methods for 1000 steps with their trained learning rates (Appendix C). We perform these computations from 100 random initializations for each gradient method, then project the result to ultrametric space using the single-linkage algorithm, which is known to coincide with tropical projection [38, 39], and record the topology of the resulting tree. The counts for each observed tree topology are presented in Tables 3 and 4.

**Table 3:** The counts of each Newick string (up to tree topology) returned by the tropical projection of a Fermat–Weber point computed via each gradient method. Each method is run for 1000 steps with trained learning rate, from 100 random initializations

Newick Tree	Adam	Adamax	CD	SGD	TD	TrAdamax	TSGD
((B,A),(((F,E),(H,G)),(D,C)));	0	0	0	0	0	0	2
(((((F,E),(H,G)),(D,C)),A),B);	0	0	0	3	0	0	0
(((((F,E),(H,G)),(D,C)),B),A);	100	100	100	97	100	100	98

**Table 4:** The counts of each Newick string (up to tree topology) returned by the tropical projection of a Fréchet means computed via each gradient method. Each method is run for 1000 steps with trained learning rate, from 100 random initializations

Newick Tree	Adam	Adamax	CD	SGD	TD	TrAdamax	TSGD
((B,A),(((F,E),(H,G)),(D,C)));	0	0	0	0	0	0	5
(((((F,E),(H,G)),(D,C)),A),B);	0	0	0	1	0	0	1
(((((F,E),(H,G)),(D,C)),B),A);	100	100	100	99	100	100	94

We first note that in the vast majority of cases, our calculations recover the tree topology of the original species tree, suggesting that for this multi-species coalescent dataset, the tropical Fermat–Weber and Frécht mean points are sufficiently good estimators for the species tree. When comparing the performance of each gradient method, we notice that the stochastic methods are the only methods to not recover the species tree topology, with Fréchet means computed via TSGD producing the highest error rate.

The relative performance of tropical Fermat–Weber and Fréchet mean points as estimators for multi-species coalescent species trees is currently an open topic of study [40]. In exploring the consistency of these estimators it world be natural for future work to also consider the relative performance of tropical gradient methods for their computation.

#### Auction model with hidden preference factors

As a final computational demonstration, we use tropical gradient methods to solve the problem of tropical linear regression using the toy auction data example given in [15]. For *N* firms and *K* products, we let $(p_{ij})_{i \le N, j \le K}$ denote the price firm *i* offers for product *j*. Assuming the offered prices are in a state of equilibrium, that is, no firm can raise their price and still win, differences in proposed price from one firm relative to another is indicative of a hidden preference factor $f_i$ in the market; that is, two firms’ offers for product *j* are considered equal when $p_{i_1j}f_{i_1} = p_{i_2j}f_{i_2}$. By performing tropical linear regression on the data $V_{ij} = \exp (-p_{ij})$, we can detect these hidden preference factors. For further details of this model and its assumptions, see [15].

For our computations, we take $N=3$ and $K=6,100$, using the same toy datasets discussed in Section 7.2 of [15], which have true hidden preference factors $f = (1, 0.8, 0.6)$. We then perform each gradient method for 1000 steps over 100 random initializations, using learning rates which have been tuned to these datasets (Appendix C). Our estimates for *f* are then given by ${\hat{f}} :=\exp (\textbf{t}_{1000}-\max (\textbf{t}_{1000}))$, such that $\max _i {\hat{f}} = 1$.

**Table 5:** The mean(± 1 std.) estimators ${\hat{f}} :=\exp (\textbf{t}_{1000}-\max (\textbf{t}_{1000}))$ for the hidden preference factors in the Auction model [15] when computed using gradient methods to solve the tropical linear regression problem. The true hidden preference factors are $f= (1,0.8,0.6)$. [15] uses the projective Krasnoselskii-Mann iteration to solve this tropical linear regression problem, which produced an estimate of $f^{\text {reg}} = (1, 0.81, 0.605)$ for the $K=6$ toy dataset

	K=6	K=100
Adam	(0.76 ± 0.33, 0.70 ± 0.32, 0.56 ± 0.21)	(0.77 ± 0.33, 0.71 ± 0.32, 0.58 ± 0.21)
Adamax	(0.76 ± 0.33, 0.70 ± 0.32, 0.56 ± 0.21)	(0.77 ± 0.33, 0.71 ± 0.32, 0.59 ± 0.21)
CD	(0.71 ± 0.43, 0.60 ± 0.42, 0.45 ± 0.30)	(0.78 ± 0.33, 0.70 ± 0.32, 0.58 ± 0.20)
SGD	(0.73 ± 0.44, 0.57 ± 0.43, 0.41 ± 0.32)	(0.77 ± 0.33, 0.70 ± 0.32, 0.58 ± 0.21)
TD	(1.00 ± 0.00, 0.81 ± 0.02, 0.60 ± 0.01)	(1.00 ± 0.02, 0.75 ± 0.08, 0.62 ± 0.05)
TrAdamax	(1.00 ± 0.00, 0.81 ± 0.00, 0.60 ± 0.00)	(0.93 ± 0.19, 0.79 ± 0.16, 0.65 ± 0.08)
TSGD	(1.00 ± 0.00, 0.82 ± 0.02, 0.60 ± 0.01)	(1.00 ± 0.04, 0.79 ± 0.04, 0.61 ± 0.01)

Table 5 shows the resulting mean estimates for these hidden preference factors when using different gradient methods to solve the tropical linear regression problem. We see that the accuracy of the tropical gradient methods vastly exceeds each of the classical gradient methods, and that these gradient methods produce particularly consistent results for the small sample problem.

These computations are limited to a single Auction model, but show promising results. For future work, it would be of interest to explore how the precision of these methods changes with sample size and dimension, as well as a running time comparison with existing methods such as the projective Krasnoselskii-Mann iteration used by [15] or combinatorial simplex algorithms [41].

## Conclusion and discussion

We have introduced the concept of tropical descent as the steepest descent method with respect to the tropical norm. We have demonstrated, both theoretically and experimentally, that it exhibits desirable properties with respect to a wide class of optimization problems on the tropical projective torus, including several key statistical problems for the analysis of phylogenetic data. As well as proposing our own framework for tropical gradient methods, this work also provides a detailed review of the varying behavior of popular gradient methods when applied to tropical statistical optimization problems.

Our theoretical results outline the relative strengths of tropical descent over classical descent for tropical location problems, but only provide global solvability for 1-sample problems. In practice, our experimental results go much further; the optimization problems studied are location problems, rather than $\triangle _{\min }$-star-quasi-convex problems, and yet tropical descent appears to consistently converge to a global minimum, not just the tropical convex hull of our dataset. It would therefore be a natural extension of this work to identify how much we can relax the $\triangle _{\min }$-star-quasi-convexity condition while guaranteeing global solvability.

In this paper we have considered tree data with 4 to 8 leaves, and samples of size 10 to 100—at this scale, we have observed (T/)SGD to be a significant sacrifice in accuracy with little benefit in computational time. Furthermore, at this scale, the relative merits of SGD versus its tropical counterpart are inconsistent; TSGD out-performs SGD when solving the linear regression problem, but performs worse when finding centrality statistics. In a similar vein, initial investigations suggest that the classical stochastic algorithm for the computation of Fréchet means—Sturm’s algorithm [42]—performs poorly for the computation of tropical Fréchet means due to the inconsistent curvature of the tropical projective torus [43]. However, for applications to larger datasets and more complex objective functions, stochastic gradient methods may become necessary. It would then be important to understand the theoretical differences between SGD and TSGD in terms of their convergence guarantees in the tropical setting and their comparative performance at different scales of data dimension.

The recent identification of tropical rational functions and ReLU neural networks [9] has inspired new avenues of research in tropical applications, such as the tropical geometry of neural networks [11, 44]. It is therefore natural to consider the possible extensions of this work to neural network training; can we identify local tropical quasi-convexity in the loss functions of ReLU neural networks, and how can we extend tropical descent to the space of tropical rational functions?

Finally, as shown by [15], the tropical linear regression problem is polynomial-time equivalent to mean payoff games—two-player perfect information games on a directed graph [45, 46]. This family of problems is of particular interest in game theory as they are known to be NP $\cap$ co-NP. In our numerical experiments, tropical gradient methods demonstrate a particularly strong performance in solving tropical linear regression, and while our methods are designed to approximate the minimum rather than compute it exactly, tropical descent may prove to be an efficient tool when solving large-scale mean payoff games.

## Figures and Tables

**Fig. 11:**
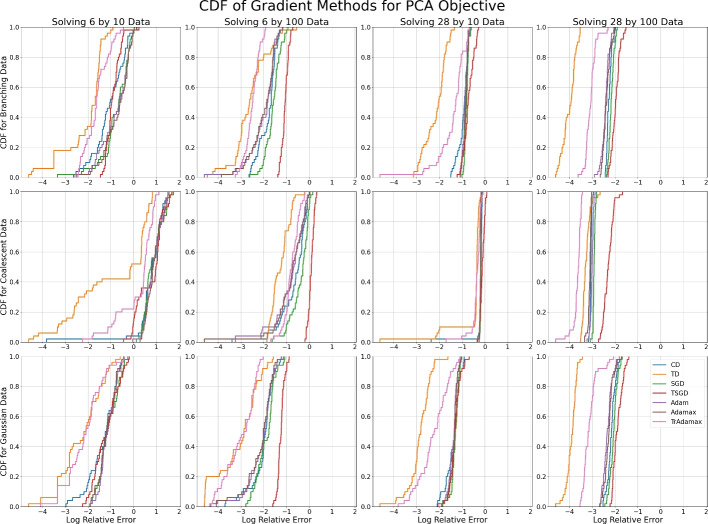
The CDF of the log relative error after 1000 steps for each gradient method with tuned learning rates across 50 random (Gaussian) initializations when minimizing the PCA objective. Each subfigure corresponds to a dataset with sample size $K=10,100$ of dimensionality $N=6,28$, sampled from a branching process, coalescent process, or a Gaussian distribution

**Fig. 12:**
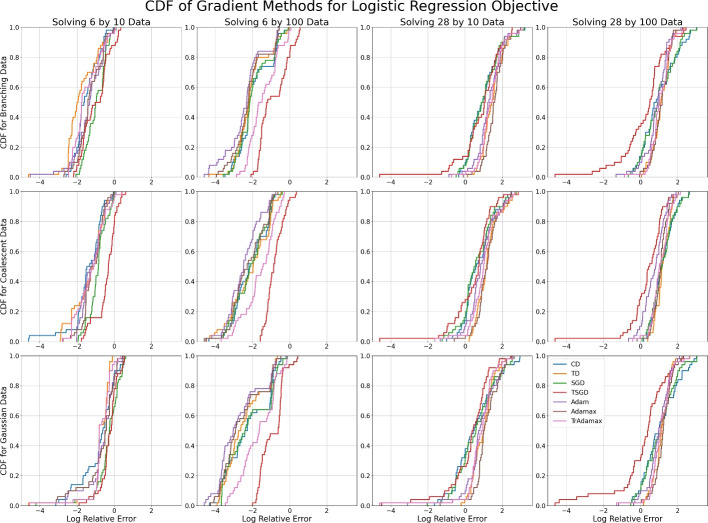
The CDF of the log relative error after 1000 steps for each gradient method with tuned learning rates across 50 random (Gaussian) initializations when minimizing the logistic regression objective. Each subfigure corresponds to a dataset with sample size $K=10,100$ of dimensionality $N=6,28$, sampled from a branching process, coalescent process, or a Gaussian distribution

**Fig. 13:**
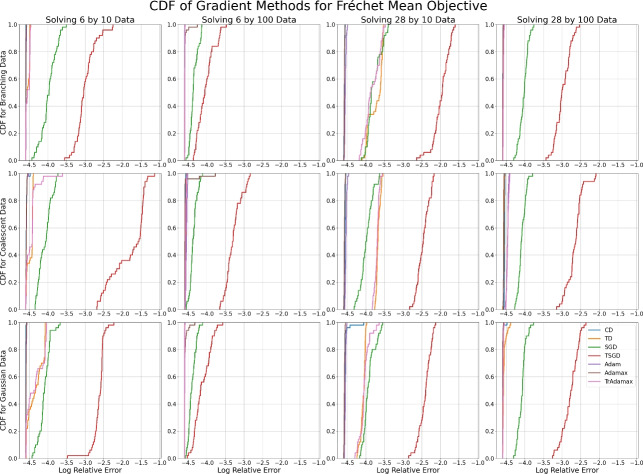
The CDF of the log relative error after 1000 steps for each gradient method with tuned learning rates across 50 random (Gaussian) initializations when minimizing the Fréchet mean objective. Each subfigure corresponds to a dataset with sample size $K=10,100$ of dimensionality $N=6,28$, sampled from a branching process, coalescent process, or a Gaussian distribution

## Appendix A PCA and logistic regression

As well as the location problems outlined in this paper, we performed the same numerical experiments with the tropical polytope PCA loss function [47] and tropical logistic regression loss function [48]. These loss functions take multiple points in $\mathbb {R}^{N}/\mathbb {R}\textbf{1}$ as argument, and we execute our gradient methods on each argument simultaneously. Figures 11 and 12 show the resulting CDF of our gradient methods for these problems. These problems do not lend themselves well to gradient methods, as the relative log error is rather large for all of our proposed methods. However, for both problems the tropical descent and TrAdamax methods are outperforming the classical methods quite consistently.

## Appendix B Methodology

The same 12 datasets are used for each experiment in Sections 3.2 and 4. These datasets live in $\mathbb {R}^{6}/\mathbb {R}\textbf{1}$ or $\mathbb {R}^{28}/\mathbb {R}\textbf{1}$; these dimensions correspond to the spaces of trees with 4 and 8 leaves respectively. Branching process datasets are generated using the R package ape [49] with exponentially distributed branch lengths—this produces data supported on the space of metric trees, i.e., the tropical Grassmannian. Coalescent data has also been generated using ape and is supported on the space of ultrametric trees, which is a tropical linear space. Gaussian data is generated using NumPy [50], and is supported on the general tropical projective torus.

In the computation of the Wasserstein projection objectives, we require a secondary dataset of different dimensionality; for each of the 12 datasets, we generate datasets of the same size and distribution, but in $\mathbb {R}^{3}/\mathbb {R}\textbf{1}$ or $\mathbb {R}^{21}/\mathbb {R}\textbf{1}$ which contain the spaces of trees with 3 and 7 leaves respectively.

We use PyTorch [30] for the computation and differentiation of each objective function. Max-tropical descent directions are used for tropical gradient methods; we therefore optimize the min-tropical location problems—Wasserstein projections and tropical linear regression—with respect to $-\textbf{t}$.

## Appendix C Learning rate tuning

The Adam algorithms—Adam, Adamax and TrAdamax—are run using the default $\beta = (0.9, 0.999)$ parameters. We then tune the learning rates used for each method; CD, TD, SGD, TSGD, Adam, Adamax, and TrAdamax. We take 10 initializations at unit intervals on a log scale in the region $[e^{-6}, e^4]$ for each problem, gradient method, and data dimensionality. We then selected the learning rate with minimal mean log relative error in each case; Tables 6, 7, 8, 9, 10, 11, 12 show the selected learning rates.

For the Auction data in Section 4.3.2, we take 10 initializations at unit intervals in the region of $[e^{-10},e^5]$ for each dataset; Table 13 shows the selected learning rates which minimize the mean log relative error.

**Table 6:** Tuned learning rates for each gradient method when solving the Fermat–Weber problem

Data Dim	Data Count	CD	TD	SGD	TSGD	Adam	Adamax	TrAdamax
6	10	0.135	0.135	0.135	0.135	0.00674	0.00674	0.00674
	100	0.135	0.135	0.135	0.135	0.00248	0.00248	0.00674
28	10	0.368	0.368	0.368	1	0.00674	0.00674	0.0183
	100	0.368	0.135	0.368	1	0.00674	0.00674	0.0183

**Table 7:** Tuned learning rates for each gradient method when solving the Fréchet mean problem

Data Dim	Data Count	CD	TD	SGD	TSGD	Adam	Adamax	TrAdamax
6	10	0.135	0.135	0.135	0.135	0.00674	0.00674	0.00674
	100	0.135	0.135	0.135	0.135	0.00674	0.00248	0.00674
28	10	0.368	0.368	0.368	1	0.00674	0.00674	0.0183
	100	0.368	0.135	0.368	1	0.00674	0.00674	0.0183

**Table 8:** Tuned learning rates for each gradient method when solving the 2-Wasserstein projection problem

Data Dim	Data Count	CD	TD	SGD	TSGD	Adam	Adamax	TrAdamax
6	10	2.72	1	1	0.135	0.135	0.368	0.368
	100	2.72	2.72	0.368	0.135	0.135	0.135	0.135
28	10	0.368	0.368	0.368	1	0.00674	0.0183	0.0183
	100	2.72	2.72	0.368	1	0.0498	0.135	0.0498

**Table 9:** Tuned learning rates for each gradient method when solving the $\infty$-Wasserstein projection problem

Data Dim	Data Count	CD	TD	SGD	TSGD	Adam	Adamax	TrAdamax
6	10	0.0498	0.135	0.0498	0.368	0.00674	0.00674	0.0183
	100	0.0498	0.135	0.135	2.72	0.00674	0.00674	0.0183
28	10	0.368	1	0.368	1	0.00674	0.0183	0.0498
	100	0.368	1	0.368	1	0.00674	0.0183	0.0498

**Table 10:** Tuned learning rates for each gradient method when solving the polytope PCA problem

Data Dim	Data Count	CD	TD	SGD	TSGD	Adam	Adamax	TrAdamax
6	10	2.72	2.72	1	1	0.135	0.135	0.368
	100	2.72	7.39	1	1	0.135	0.135	1
28	10	7.39	7.39	0.368	1	0.0183	0.0183	0.135
	100	2.72	7.39	0.368	1	0.0498	0.0498	0.0498

**Table 11:** Tuned learning rates for each gradient method when solving the logistic regression problem

Data Dim	Data Count	CD	TD	SGD	TSGD	Adam	Adamax	TrAdamax
6	10	7.39	2.72	1	2.72	0.368	1	0.0498
	100	1	0.368	1	2.72	0.00248	0.00248	0.00248
28	10	1	0.368	1	2.72	0.00248	0.00248	0.00674
	100	1	0.368	1	7.39	0.00248	0.00248	0.00674

**Table 12:** Tuned learning rates for each gradient method when solving the linear regression problem

Data Dim	Data Count	CD	TD	SGD	TSGD	Adam	Adamax	TrAdamax
6	10	0.368	0.135	0.368	0.135	0.00248	0.0183	0.00674
	100	0.135	0.135	0.368	0.0498	0.00248	0.00248	0.00674
28	10	0.368	0.0498	0.0498	0.135	0.00248	0.00248	0.0183
	100	1	0.368	1	7.39	0.0498	0.0498	0.0498

**Table 13:** Tuned learning rates for each gradient method when solving the linear regression problem with Auction data

Data Dim	Data Count	CD	TD	SGD	TSGD	Adam	Adamax	TrAdamax
3	6	2.72	0.368	7.39	0.0498	0.000912	0.000912	0.0183
	100	0.0183	2.72	0.0183	0.0498	0.000912	0.000912	0.00248

## Appendix D Run times

Experiments were implemented on a AMD EPYC 7742 node using a single core, 96 GB. Table 14 shows the average time taken per initialization for each gradient method and optimization problem.

**Table 14:** Average run times for 1000 steps of each gradient method when minimizing each objective function

Stats Prob	CD	TD	SGD	TSGD	Adam	Adamax	TrAdamax
Fermat–Weber	0.480	0.520	0.435	0.499	0.391	0.460	0.464
Fréchet Mean	0.347	0.363	0.301	0.342	0.294	0.422	0.370
2-Wasserstein Projection	2.739	2.134	2.392	2.209	2.341	2.246	2.272
$\infty$-Wasserstein Projection	2.947	3.049	2.772	2.565	2.501	3.162	2.807
Polytope PCA	1.117	1.081	1.014	1.052	1.037	1.174	1.029
Logistic Regression	0.987	1.015	0.962	0.934	0.943	1.084	0.943
Linear Regression	0.494	0.499	0.386	0.420	0.386	0.537	0.433

## Appendix E Further figures

### E.1 Fréchet means

Here we include the results for experiments run for the computation of Fréchet means. (Table 15, Fig. 13).

**Table 15:** The mean log relative error of each gradient method after 1000 steps with tuned learning rates for branching process, coalescent process, and Gaussian datasets of size $K=10,100$ in $\mathbb {R}^{6}/\mathbb {R}\textbf{1}$, $\mathbb {R}^{28}/\mathbb {R}\textbf{1}$ when minimizing the Fréchet mean objective function. The minimal mean errors for each dataset are in bold

Data	CD	TD	SGD	TSGD	Adam	Adamax	TrAdamax
6$\times$10 Branching Data	$\varvec{-}$**4.59**	−4.54	−3.98	−2.99	−4.58	$\varvec{-}$**4.59**	−4.53
6$\times$10 Coalescent Data	$\varvec{-}$**4.58**	−4.47	−4.07	−1.82	−4.57	$\varvec{-}$**4.58**	−4.45
6$\times$10 Gaussian Data	$\varvec{-}$**4.59**	−4.33	−4.09	−2.63	−4.58	$\varvec{-}$**4.59**	−4.35
6$\times$100 Branching Data	$\varvec{-}$**4.59**	$\varvec{-}$**4.59**	−4.35	−4.03	$\varvec{-}$**4.59**	−4.58	$\varvec{-}$**4.59**
6$\times$100 Coalescent Data	$\varvec{-}$**4.57**	−4.56	−4.36	−3.29	−4.55	−4.56	$\varvec{-}$**4.57**
6$\times$100 Gaussian Data	−4.59	$\varvec{-}$**4.60**	−4.39	−4.12	−4.59	−4.58	−4.59
28$\times$10 Branching Data	$\varvec{-}$**4.58**	−3.73	−3.78	−1.98	−4.55	$\varvec{-}$**4.58**	−3.83
28$\times$10 Coalescent Data	−4.56	−3.67	−3.98	−2.46	−4.53	$\varvec{-}$**4.57**	−3.70
28$\times$10 Gaussian Data	−4.55	−4.07	−3.91	−2.41	−4.55	$\varvec{-}$**4.57**	−4.04
28$\times$100 Branching Data	$\varvec{-}$**4.59**	−4.58	−4.03	−2.98	−4.58	$\varvec{-}$**4.59**	−4.57
28$\times$100 Coalescent Data	−4.54	−4.56	−4.08	−2.66	−4.47	$\varvec{-}$**4.57**	−4.46
28$\times$100 Gaussian Data	$\varvec{-}$**4.59**	−4.56	−4.08	−2.78	$\varvec{-}$**4.59**	$\varvec{-}$**4.59**	−4.58

### E.2 Wasserstein projections

From Tables 16 and 17, we see that when solving the 2-Wasserstein projection problem, tropical methods are optimal in all cases other than $28\times 10$ coalescent data. For the $\infty$-Wasserstein projection problem in small dimensions, tropical descent outperforms other methods by some margin, while the comparative performances are less consistent for the $\infty$-Wasserstein projection problem in high dimensions.

**Table 16:** The mean log relative error of each gradient method after 1000 steps with tuned learning rates for branching process, coalescent process, and Gaussian datasets of size $K=10,100$ in $\mathbb {R}^{6}/\mathbb {R}\textbf{1}$, $\mathbb {R}^{28}/\mathbb {R}\textbf{1}$ when minimizing the 2-Wasserstein projection objective function. The minimal mean errors for each dataset are in bold

Data	CD	TD	SGD	TSGD	Adam	Adamax	TrAdamax
6$\times$10 Branching Data	−3.02	$\varvec{-}$**4.09**	−2.16	−2.11	−2.95	−3.00	−3.89
6$\times$10 Coalescent Data	−3.71	−3.92	−2.44	−3.13	−3.78	−3.68	$\varvec{-}$**4.12**
6$\times$10 Gaussian Data	−3.59	−3.83	−2.87	−2.75	−3.61	−3.57	$\varvec{-}$**3.89**
6$\times$100 Branching Data	−3.44	$\varvec{-}$**4.24**	−2.91	−1.60	−3.79	−3.79	−4.02
6$\times$100 Coalescent Data	−4.26	$\varvec{-}$**4.55**	−3.27	−2.58	−4.39	−4.45	−4.50
6$\times$100 Gaussian Data	−3.69	−4.17	−3.33	−2.73	−3.78	−3.78	**−4.18**
28$\times$10 Branching Data	−3.39	−4.31	−3.16	−2.03	−3.53	−3.55	$\varvec{-}$**4.32**
28$\times$10 Coalescent Data	−4.11	−3.79	−3.39	−2.42	$\varvec{-}$**4.17**	−4.12	−3.80
28$\times$10 Gaussian Data	−3.43	$\varvec{-}$**3.91**	−3.21	−2.15	−3.50	−3.44	−3.83
28$\times$100 Branching Data	−3.61	$\varvec{-}$**4.55**	−3.37	−2.65	−3.85	−3.85	−4.51
28$\times$100 Coalescent Data	−4.11	$\varvec{-}$**4.57**	−3.89	−3.32	−4.21	−4.19	−4.56
28$\times$100 Gaussian Data	−3.66	$\varvec{-}$**4.52**	−3.41	−2.61	−3.82	−3.80	−4.49

**Table 17:** The mean log relative error of each gradient method after 1000 steps with tuned learning rates for branching process, coalescent process, and Gaussian datasets of size $K=10,100$ in $\mathbb {R}^{6}/\mathbb {R}\textbf{1}$, $\mathbb {R}^{28}/\mathbb {R}\textbf{1}$ when minimizing the $\infty$-Wasserstein projection objective function. The minimal mean errors for each dataset are in bold

Data	CD	TD	SGD	TSGD	Adam	Adamax	TrAdamax
6$\times$10 Branching Data	−1.63	$\varvec{-}$**4.32**	−0.57	−0.91	−1.68	−1.69	$\varvec{-}$**4.32**
6$\times$10 Coalescent Data	−4.00	$\varvec{-}$**4.47**	−0.99	−1.43	−4.03	−4.04	−4.46
6$\times$10 Gaussian Data	−3.77	$\varvec{-}$**4.42**	−1.76	−3.33	−3.76	−3.75	−4.40
6$\times$100 Branching Data	−3.35	$\varvec{-}$**4.48**	−1.94	−0.95	−3.34	−3.36	−4.47
6$\times$100 Coalescent Data	−3.85	$\varvec{-}$**4.51**	−2.06	−2.21	−3.84	−3.85	$\varvec{-}$**4.51**
6$\times$100 Gaussian Data	−3.63	$\varvec{-}$**4.40**	−1.61	−1.22	−3.70	−3.63	−4.36
28$\times$10 Branching Data	−3.68	−3.53	−2.22	−1.17	$\varvec{-}$**4.11**	−3.93	−3.17
28$\times$10 Coalescent Data	−4.22	−3.95	−2.76	−1.92	$\varvec{-}$**4.42**	−4.36	−3.94
28$\times$10 Gaussian Data	−4.02	−3.14	−2.13	−0.95	$\varvec{-}$**4.43**	−4.30	−2.85
28$\times$100 Branching Data	−3.46	−3.58	−1.37	−1.19	$\varvec{-}$**3.68**	−3.59	−3.39
28$\times$100 Coalescent Data	−4.41	−3.92	−2.09	−2.00	$\varvec{-}$**4.54**	−4.51	−3.81
28$\times$100 Gaussian Data	−2.98	$\varvec{-}$**3.30**	−1.64	−1.61	−3.07	−3.04	−2.80
